# Relationship between traditional risk factors for hypertension and systolic blood pressure in the Tohoku Medical Megabank Community-based Cohort Study

**DOI:** 10.1038/s41440-024-01582-1

**Published:** 2024-02-29

**Authors:** Masato Takase, Naoki Nakaya, Kozo Tanno, Mana Kogure, Rieko Hatanaka, Kumi Nakaya, Ippei Chiba, Ikumi Kanno, Kotaro Nochioka, Naho Tsuchiya, Tomohiro Nakamura, Takumi Hirata, Taku Obara, Mami Ishikuro, Yuka Kotozaki, Akira Uruno, Tomoko Kobayashi, Eiichi N. Kodama, Yohei Hamanaka, Masatsugu Orui, Soichi Ogishima, Satoshi Nagaie, Hideki Ohmomo, Nobuo Fuse, Junichi Sugawara, Atsushi Shimizu, Yoko Izumi, Shinichi Kuriyama, Atsushi Hozawa, the ToMMo investigators

**Affiliations:** 1 Graduate School of Medicine, Tohoku University, Aoba-ku, Sendai, Miyagi Japan; 2 Tohoku Medical Megabank Organization, Tohoku University, Aoba-ku, Sendai, Miyagi Japan; 3 Iwate Medical Megabank Organization, Iwate Medical University, Kamaishi, Iwate Japan; 4 School of Medicine, Iwate Medical University, Morioka, Iwate Japan; 5 Tohoku University Hospital, Tohoku University, Aoba-ku, Sendai, Miyagi Japan; 6 Kyoto Women’s University, Kyoto, Japan; 7 Institute for Clinical and Translational Science, Nara Medical University, Shijo-cho, Kashihara, Nara, Japan; 8 International Research Institute of Disaster Science, Tohoku University, Aoba-ku, Sendai, Miyagi Japan; 9 Suzuki Memorial Hospital, Satonomori, Iwanumashi, Miyagi Japan

**Keywords:** Blood pressure, Epidemiology, Gamma-glutamyl transferase, Hypertension, Risk factor

## Abstract

Risk factors for hypertension have been emphasized in the Japanese Society of Hypertension Guidelines for the Management of Hypertension. However, large-scale studies on the association of smoking, potassium excretion, and gamma-glutamyl transferase level with BP in the Japanese population are limited. We conducted a cross-sectional study to examine the association between hypertension risk factors and systolic blood pressure in the Tohoku Medical Megabank Community-based Cohort Study (23,446 men and 38,921 women aged ≥20 years). A model adjusted for age, body mass index, smoking status, drinking status, estimated daily salt intake, potassium excretion, (or urinary sodium-to-potassium ratio), gamma-glutamyl transferase, physical activity, education level, status of damage to homes during the Great East Japan Earthquake, and residential areas was used. The average age and systolic blood pressure were 62.5 (10.3) years for men and 59.6 (11.3) years for women, 128.9 (16.7) mmHg for men and 124.7 (17.5) mmHg for women, respectively. Body mass index estimated daily salt intake, urinary sodium-to-potassium ratio and gamma-glutamyl transferase levels were positively associated with systolic blood pressure. Compared with never-drinkers, current drinkers who consumed 23–45 g/day and ≥46.0 g/day had significantly increased systolic blood pressure. Conversely, current smokers (1-10 cigarettes/day and 11-20 cigarettes/day) were inversely associated with systolic blood pressure compared to never-smokers. Overall, systolic blood pressure was associated with gamma-glutamyl transferase and hypertension risk factors, including body mass index, alcohol consumption, estimated daily salt intake, urinary sodium-to-potassium ratio, and potassium excretion. Our findings support the notion that lifestyle modifications should be attempted to prevent hypertension.

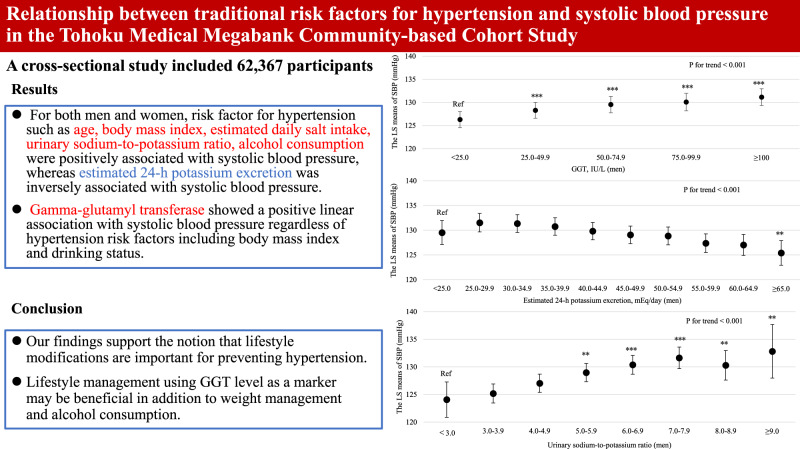

## Introduction

Several risk factors for hypertension, such as obesity, salt intake, potassium intake, physical activity, alcohol consumption, and smoking habits, have been highlighted in various guidelines, including the Japanese Society of Hypertension Guidelines for the Management of Hypertension (JSH 2019) [1–3]. To date, many epidemiological studies have examined the association between blood pressure (BP) and the risk factors for hypertension in the Japanese population. The National Integrated Project for Prospective Observation of Noncommunicable Disease and its Trends in the Aged (NIPPON DATA) showed that body mass index (BMI), alcohol consumption, and dietary salt intake were positively associated with the prevalence of hypertension [4–6]. Additionally, an investigation of Japanese in the International Study of Electrolyte Excretion and Blood Pressure (INTERSOLT) found that sodium excretion, BMI, and heavy alcohol consumption were associated with high BP, whereas potassium excretion was inversely associated with BP [7].

However, several issues remain to be resolved. First, the association between smoking and BP remains unclear. Previous epidemiological studies have demonstrated that smoking is positively associated with BP [8–12], whereas other studies are not statistically significant or are inversely associated [13–17]. Second, large-scale epidemiological cohort studies including 21 countries but does not include Japan have assessed the relationship between sodium and potassium excretion and BP and have shown the slope of the relationship between sodium excretion and BP [18–23]. Although Japan is well known for its high salt intake [24], the slope of the relationship between potassium excretion and BP are unclear. Third, several prospective cohort studies and two meta-analyses have shown that the level of serum gamma-glutamyl transferase (GGT), which is used as a marker of excessive alcohol consumption and visceral fat, particularly in hepatic steatosis [25, 26], can predict future hypertension [27–32]. However, there are only three small epidemiological studies (n < 1,600) that have investigated whether GGT level are associated with BP, regardless of obesity and drinking status, in the Japanese population [27, 33, 34]. Additionally, two of those previous studies included only men [27, 33], and the other study did not stratify by sex even though GGT level vary widely by sex [34]. Supposing that GGT is associated with BP independent of conventional risk factors, including obesity and alcohol consumption, it may be necessary to consider individuals with elevated GGT levels to be at high risk for hypertension. Therefore, to better understand the association between GGT as well as hypertension risk factors and BP among Japanese population, a large-scale Japanese population-based study is required.

The Tohoku Medical Megabank Project (TMM), which was conducted by the Tohoku University Tohoku Medical Megabank Organization and Iwate Medical University Tohoku Medical Megabank Organization, was established to assess the long-term impact of the Great East Japan Earthquake (GEJE) on disaster victims [35–37]. The TMM Community-Based Cohort Study (TMM CommCohort Study) [37], were established, and information on genes, lifestyle, blood, and urinary parameters was obtained. This large-scale population-based cohort studies can reveal aspects of the association between BP and not only conventional risk factors for hypertension, including those mentioned in JSH 2019, but also other risk factors, such as GGT. A detailed description of the association between risk factors and SBP in a large population would provide a basis for better understanding of that relationship. Therefore, in this study, we examined the association between systolic blood pressure (SBP) and hypertension risk factors by sex in more than 62,000 Japanese participants.

Point of view

- **Clinical relevance**
  SBP is associated with the risk factors including daily salt intake, potassium excretion, urinary sodium-to-potassium ratio, BMI and alcohol intake, for hypertension, as described in JSH 2019.
- **Future direction**
  A prospective cohort study to better understand the relationship between hypertension risk factors and blood pressure, including using objective measures of physical activity are warranted.
- **Consideration for the Asian population**
  Asian cuisines are salt-heavy, thus, recommending salt reduction and potassium intake using a urinary sodium-to-potassium ratio as an indicator may be useful for blood pressure management.

## Methods

### Study design and population

We conducted a cross-sectional study using data from the TMM CommCohort Study, which has been previously described [35, 37]. The source population for this study comprised males and females aged ≥20 years who were living in the Miyagi and Iwate prefectures in northeastern Japan. All participants were recruited between May 2013 and March 2016. Written informed consent was obtained from all participants. The Institutional Review Board of the Tohoku Medical Megabank Organization approved this study (approval number:2022-4-047; approval date: June 30, 2022).

Overall, 67,355 participants were initially enrolled in a baseline survey of municipal health checkups. Of them, we excluded 4,988 for the following reasons: (1) those who withdrew from the study by November 16, 2021, and lacked a self-reported questionnaire, and (2) those with missing data on SBP, diastolic blood pressure (DBP), height, weight, urinary creatinine, estimated urinary 24-h sodium excretion and potassium excretion, and GGT. Finally, we analyzed data for 62,367 participants.

### Measurements

Information on BP was obtained through municipal health checkups. As directed by the Ministry of Health, Labor and Welfare, BP was measured twice and the average of the two measurements were used. One measurement was also acceptable, depending on the situation at the municipal health-checkup site. BP was measured on the right upper arm after urinating and resting in a sitting position for at least 5 min, avoiding conditions that would affect BP measurement, such as exercise, eating, smoking [38]. Age was determined during the visit to the municipal health checkup sites. BMI was calculated as weight (kg) divided by the square of height (m). Blood samples were collected at the municipal health checkup venues. Additionally, the serum GGT levels were measured using an enzymatic method. Casual spot urine samples were collected from all participants. Estimates of the 24-h urinary excretion of sodium and potassium and the estimated daily salt intake from spot urine samples were calculated using the Tanaka formula [39]. The urinary sodium-to-potassium ratio (Na/K ratio) was calculated by estimated of the 24-h urinary sodium excretion divided to estimates of the 24-h urinary potassium excretion. We used a self-report questionnaire to assess demographic characteristics, status of damage due to the GEJE, smoking status, drinking status, education level, physical activity, and treatment for hypertension. Smoking status was classified into the following six categories: never-smokers (had smoked <100 cigarettes in their lifestyle), ex-smokers (had smoked ≥100 cigarettes in their lifetime and were not current smokers), current smoker (1–9, 10–19, or ≥20 cigarettes a day), and unknown [40]. Educational level was classified into the following four categories: below high school, vocational school or junior college or technical college, university or graduate school, and unknown. Furthermore, the damage to homes during the GEJE was classified into the following seven groups: destroyed, large-scale particle collapse, partially destroyed, partially damaged, no damage, do not live in affected areas, and unknown. Alcohol type was classified into the following six categories: sake, distilled spirits, shochu-based beverages, beer, whiskey, and wine. Moreover, the frequency of alcohol consumption was classified into the following six categories: almost never, 1–3 days/month, 1–2 days/week, 3–4 days/week, 5–6 days/week, and daily. The quantity of ethanol consumed was calculated by multiplying the type of alcohol consumed by the frequency and volume of consumption [41, 42]. Drinking status was classified into the following six categories: never drinkers (had consumed little or no alcohol or were constitutionally incapable of alcohol consumption), ex-drinkers (had stopped drinking alcohol), current drinkers (<23 g, 23.0–45.9, or ≥46.0 g/day), and unknown. The average frequency (times/week) and duration (min/time) of normal walking, brisk walking, moderate-intensity exercise, and hard-intensity exercise during leisure time were determined using a self-reported questionnaire. Metabolic equivalents (METs) were assigned for each physical activity [43]. The amount of leisure-time physical activity (MET-min/week) was calculated by multiplying the corresponding METs score, duration, and frequency.

### Statistical analysis

All analysis was stratified by sex because the distribution of SBP and conventional risk factors vary by sex. Data are presented as mean (standard deviation [SD]) or median (interquartile range [IQR]) for continuous variables and as number (percentage) for categorical variables. The association between risk factors for hypertension and SBP was examined using an analysis of covariance. The least square (LS) means of SBP and corresponding 95% confidence intervals (CIs) are presented. The multivariable-adjusted models included age (per 1-year increment), BMI (per 1-kg/m^2^ increment), drinking status (never-drinker, ex-drinker, and current drinker <23 g, 23.0–45.9 g, ≥46.0 g/day, and unknown), GGT (per 25.0-IU/L increment), estimated daily salt intake (per 1-g/day increment), estimated 24-h potassium excretion (per 5-mEq/day increment), smoking status (never-smoker, ex-smokers, current smoker [1–9, 10–19, or ≥20 cigarettes a day] and unknown), physical activity (per 50-METs-min/week increment), education status (below high school, vocational school or junior college or technical college, university or graduate school, and unknown), damage to the home during the GEJE (completely destroyed, large scale partial collapse, partially destroyed, no damage, do not live in the affected area, and unknown), and residential area (Miyagi and Iwate). To examine the association between SBP and Na/K ratio, we analyzed the above multivariable adjusted model by replacing estimated salt intake and estimated 24-h potassium excretion with Na/K ratio. For continuous variables, including age, BMI, estimated daily salt intake, potassium excretion, Na/K ratio, GGT, and leisure-time METs, we further calculated the *p*-values to analyze linear trends by scoring the categories and entering the number as a continuous term in the regression model. The results were compared for each independent variable using Dunnett’s test with the following categories as references: age <31 years, BMI < 19.0 kg/m^2^, never-drinker, GGT < 25.0 IU/L, estimated daily salt intake <6.0 g/day, potassium excretion <20.0 mEq/day, Na/K ratio <3.0, never-smoker, physical activity <50 METs-min/week. We also conducted a sensitivity analysis excluding participants who were treated for hypertension because individuals undergoing treatment for hypertension may have modified their lifestyle. Additionally, we conducted a stratified analysis by presence or absent obesity ( ≥ 25.0 kg/m^2^) defined as World Health Organization criteria for Japanese individuals [44].

Statistical significance was set at *p* < 0.05. All analyses were performed using SAS version 9.4 for Windows (SAS Inc., Cary, NC, USA).

## Results

### Characteristics of the study population

In this study, for men, the mean (SD) values for age, BMI, SBP, DBP, estimated sodium excretion, potassium excretion, urinary Na/K ratio and daily salt intake were 62.5 (10.3) years, 24.1 (3.2) kg/m^2^, 128.9 (16.7) mmHg, 78.3 (10.1) mmHg, 169.9 (39.1) mEq/day, 41.9 (9.3) mEq/day, 4.2 (1.0) and 9.5 (2.3) g/day, respectively. The median (IQR) GGT and METs were 32.0 (22.0–55.0) and 0.0 (0.0–67.5), respectively. The proportions (%) of treatment for hypertension, never-drinkers, and never smokers were 32.8%, 21.2% and 25.8%, respectively (Supplemental Table 1).

For women, the mean (SD) values for age, BMI, SBP, DBP, estimated sodium excretion, potassium excretion, urinary Na/K ratio and daily salt intake were 59.6 (11.3) years, 23.1 (3.7) kg/m^2^, 124.7 (17.5) mmHg, 73.8 (10.3) mmHg, 165.2 (38.5) mEq/day, 42.0 (9.7) mEq/day, 4.0 (1.0) and 9.2 (2.2) g/day, respectively. The median (IQR) GGT and METs were 18.0 (14.0–26.0) and 9.0 (0.0–96.4), respectively. The proportions (%) of treatment for hypertension, never-drinkers, and never-smokers were 23.3%, 63.4% and 82.8%, respectively (Supplemental Table 1).

### Associations between risk factors for hypertension and SBP

**Fig. 1:**
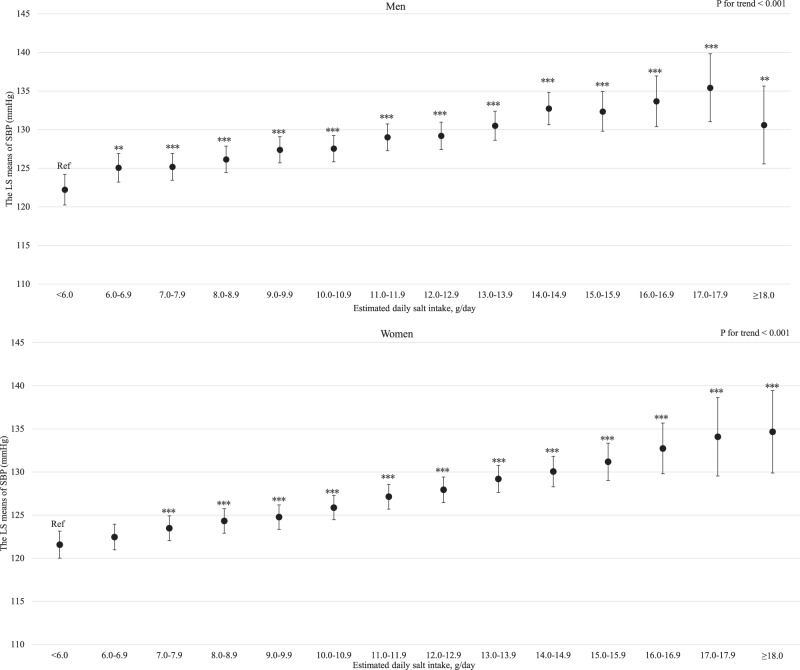
Association between estimated daily salt intake and SBP (in the model adjusted for age, BMI, GGT, drinking status, smoking status, estimated 24-h potassium excretion, physical activity, education status, damage to the home during the GEJE, and residential area). *p* for difference was derived from Dunnett’s test using estimated daily salt intake <6.0 g/day as the reference. Bars represent 95% confidence intervals. *p* for difference is shown as follows: *p < 0.05, **p < 0.01, and ***p < 0.001. *p* for trend was calculated by scoring the estimated daily salt intake categories and entering the number as a continuous term in the regression model. BMI body mass index, GGT gamma-glutamyl transferase, GEJE Great East Japan Earthquake, SBP systolic blood pressure

**Fig. 2:**
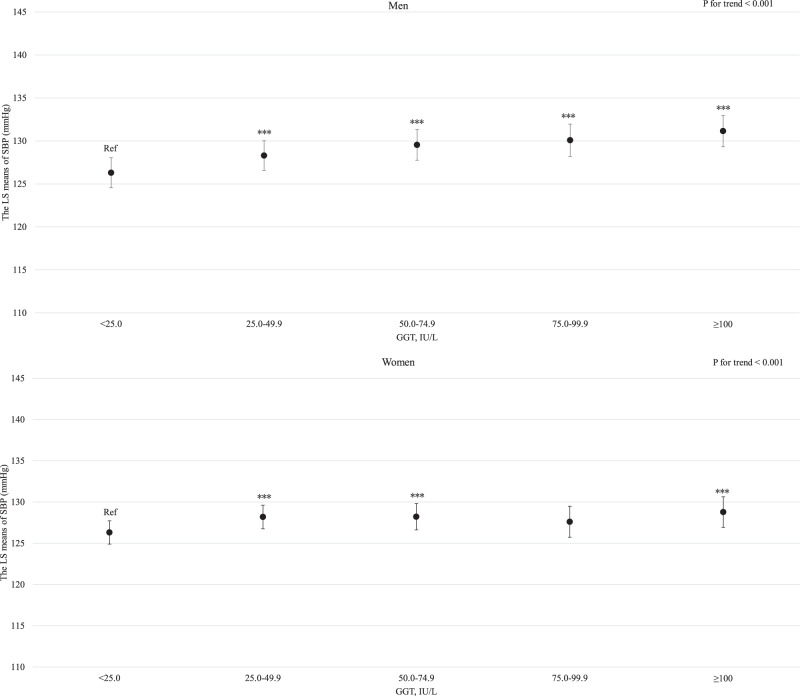
Association between GGT levels and SBP (in the model adjusted for age, BMI, estimated daily salt intake, drinking status, smoking status, estimated 24-h potassium excretion, physical activity, education status, damage to the home during the GEJE, and residential area). *p* for difference was derived from Dunnett’s test using GGT < 25.0 IU/L as the reference. Bars represent 95% confidence intervals. *p* for difference is shown as follows: *p < 0.05, **p < 0.01, and ***p < 0.001. *p* for trend was calculated by scoring the GGT categories and entering the number as a continuous term in the regression model. BMI body mass index, GGT gamma-glutamyl transferase, GEJE Great East Japan Earthquake, SBP systolic blood pressure

**Fig. 3:**
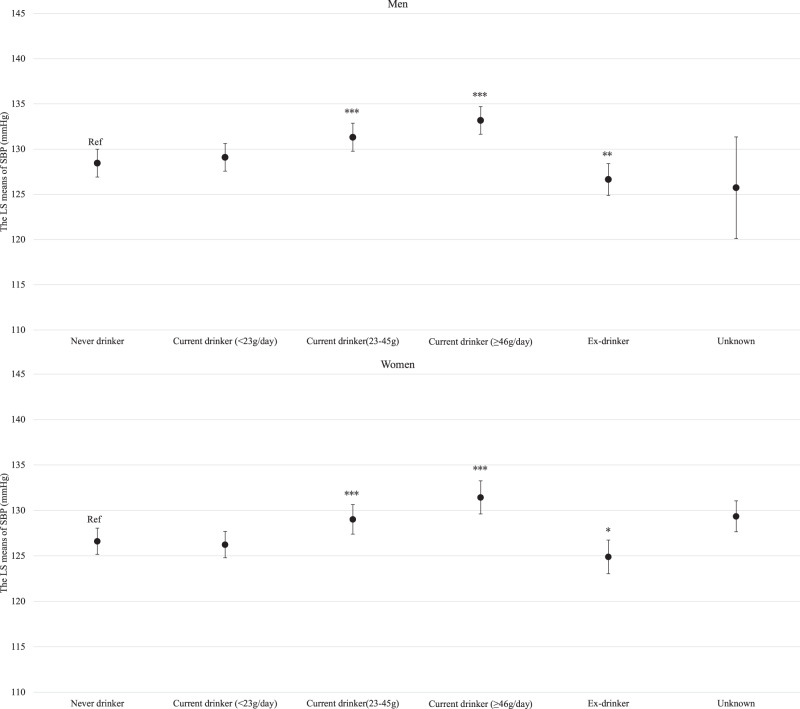
Association between drinking status and SBP (in the model adjusted for age, BMI, estimated daily salt intake, GGT, smoking status, estimated 24-h potassium excretion, physical activity, education status, damage to the home during the GEJE, and residential area). *p* for difference was derived from Dunnett’s test using never drinker as the reference. Bars represent 95% confidence intervals. *p* for difference is shown as follows: *p < 0.05, **p < 0.01, and ***p < 0.001. BMI body mass index, GGT gamma-glutamyl transferase, GEJE Great East Japan Earthquake, SBP systolic blood pressure

**Fig. 4:**
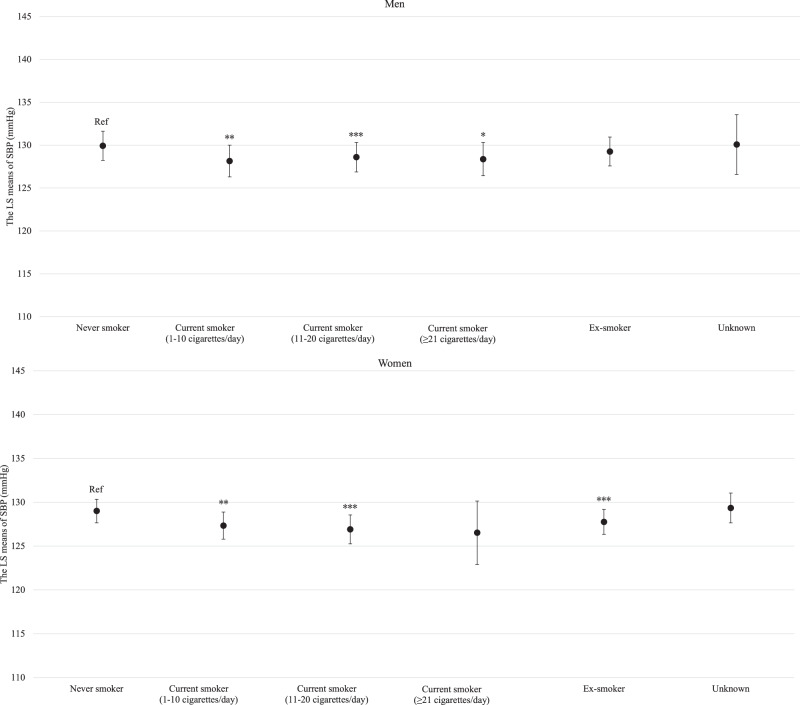
Association between smoking status and SBP (in the model adjusted for age, BMI, estimated daily salt intake, GGT, drinking status, estimated 24-h potassium excretion, physical activity, education status, damage to the home during the GEJE, and residential area). *p* for difference was derived from Dunnett’s test using never smoke as the reference. Bars represent 95% confidence intervals. *p* for difference is shown as follows: *p < 0.05, **p < 0.01, and ***p < 0.001. BMI body mass index, GGT gamma-glutamyl transferase, GEJE Great East Japan Earthquake, SBP systolic blood pressure

**Fig. 5:**
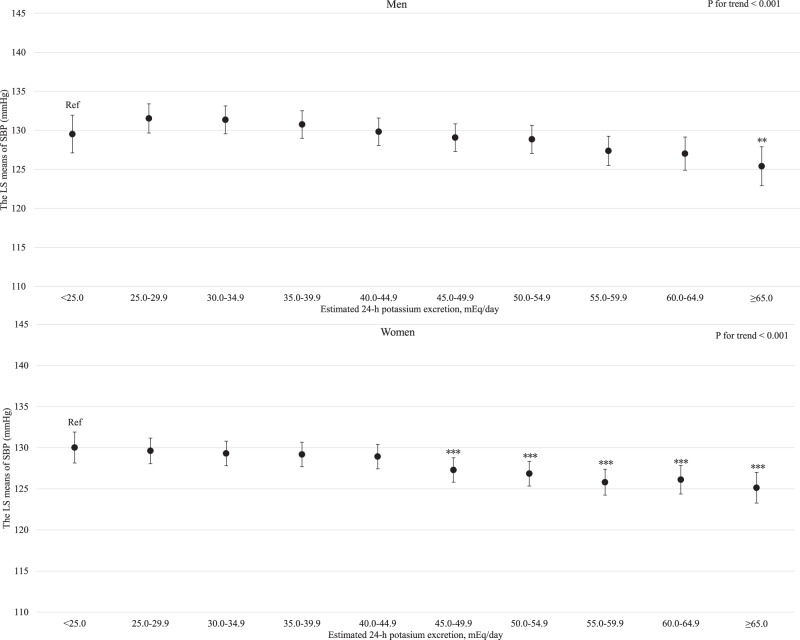
Association between estimated 24-h potassium excretion and SBP (in the model adjusted for age, BMI, estimated daily salt intake, GGT, drinking status, smoking status, physical activity, education status, damage to the home during the GEJE, and residential area). *p* for difference was derived from Dunnett’s test using estimated 24-h potassium excretion <25.0 mEq/day as the reference. Bars represent 95% confidence intervals. *p* for difference is shown as follows: *p < 0.05, **p < 0.01, and ***p < 0.001. *p* for trend was calculated by scoring the estimated 24-h potassium excretion categories and entering the number as a continuous term in the regression model. BMI body mass index, GGT gamma-glutamyl transferase, GEJE Great East Japan Earthquake, SBP systolic blood pressure

**Fig. 6:**
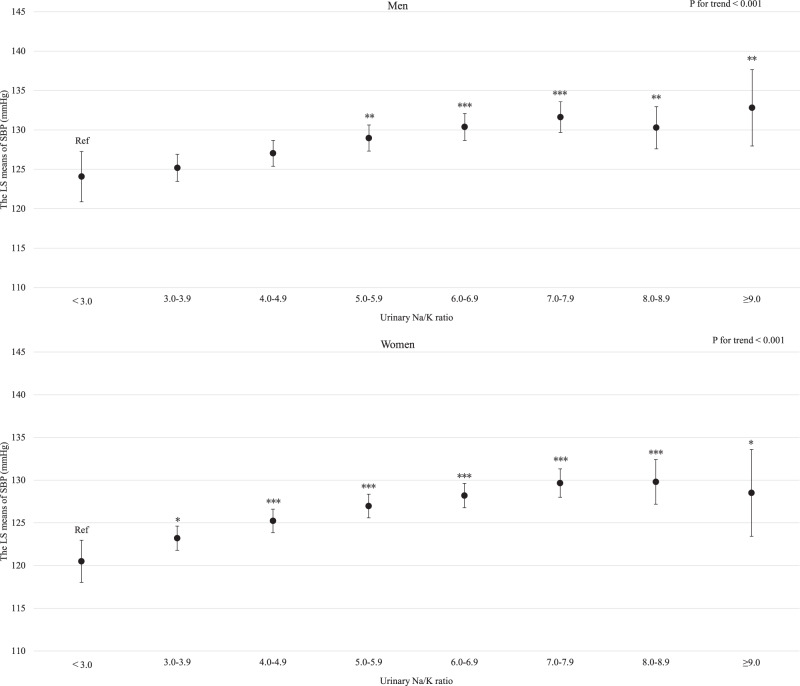
Association between urinary Na/K ratio and SBP (in the model adjusted for age, BMI, GGT, drinking status, smoking status, physical activity, education status, damage to the home during the GEJE, and residential area). *p* for difference was derived from Dunnett’s test using urinary Na/K ratio <3.0 as the reference. Bars represent 95% confidence intervals. *p* for difference is shown as follows: *p < 0.05, **p < 0.01, and ***p < 0.001. *p* for trend was calculated by scoring the urinary Na/K categories and entering the number as a continuous term in the regression model. BMI body mass index, GGT gamma-glutamyl transferase, GEJE Great East Japan Earthquake, Na/K ratio sodium to potassium ratio, SBP systolic blood pressure

For both men and women, age was clearly and linearly associated with increased SBP (*P* for linear trends <0.001; Supplemental Fig. 1). Similarly, BMI and estimated daily salt intake were linearly associated with an increased SBP even after adjusting for hypertension risk factors (Supplemental Fig. 2 and Fig. 1 for BMI and estimated daily salt intake, respectively). For both men and women, GGT showed a positive linear association with SBP regardless of hypertension risk factors, including BMI and drinking status (*P* for linear trend <0.001; Fig. 2). Compared with never-drinkers, current drinkers who consumed ethanol ≥23 g/day had significantly increased SBP; however, current drinkers who consumed ethanol <23 g/day exhibited not significantly increased SBP in men and women. However, ex-drinkers had significantly decreased SBP in both men and women (Fig. 3). The current smokers who smoked 1–10 cigarettes per day and 11–20 cigarettes per day had a significantly lower SBP than never smokers for both men and women (Fig. 4). Estimated 24-h potassium excretion was inversely associated with SBP even after considering estimated daily salt intake (Fig. 5). The urinary Na/K ratio was linearly and positively associated with SBP among men and women (Fig. 6). Leisure time physical activity were not significantly associated with SBP in men and women (Supplemental Fig. 3).

Even among participants without treatment for hypertension, the traditional risk factors for hypertension including age, BMI, estimated daily salt intake, Na/K ratio, current drinkers who consumed ethanal >23 g/day was positively associated with SBP. Conversely, the estimated potassium excretion was inversely associated with SBP (Supplemental Figs. 4–12). Additionally, although we conducted a stratified analysis by obesity, the results were substantially unchanged regardless of presence/absence obesity (Supplemental Table 2).

## Discussion

In this cross-sectional study, the association between risk factors for hypertension and SBP was analyzed using large-scale Japanese cohort study data. Our study showed that higher values of age, BMI, estimated daily salt intake, urinary Na/K ratio and GGT were positively associated with SBP, whereas estimated 24-h potassium excretion was inversely correlated with SBP for both men and women. For both men and women, current drinkers who consumed ≥23.0 g/day of ethanol had a higher SBP than never-drinkers. Conversely, compared with never-smokers, current smokers 1–10 cigarettes per day and 11–20 cigarettes per day had a significantly but slightly lower SBP in men and women. Physical activity calculated using self-reported questions were not associated with SBP for both men and women.

Several risk factors for hypertension, including salt reduction, potassium intake, physical activity, weight reduction, and drinking restrictions, have been highlighted in national and international hypertension guidelines [1–3]. For example, the JSH 2019 recommends the management of the abovementioned risk factors not only for the progression but also for the prevention of hypertension [1]. We observed that the risk factors for hypertension, including age, BMI, estimated daily salt intake, urinary Na/K ratio and alcohol consumption, were associated with elevated SBP, whereas the estimated 24-h potassium excretion was inversely associated with SBP, which is consistent with the corresponding data in JSH 2019 [1]. Previous studies have demonstrated that urinary sodium excretion, salt intake and Na/K ratio were positively associated with BP, whereas urinary potassium excretion was inversely associated with BP [18–23, 45]. Although these studies showed the slope of the association between sodium excretion and BP [18–23], those illustrating the slope of the association between potassium excretion and BP are limited among Asian population [22, 23]. We demonstrated and reconfirmed the linear relationship between salt intake, Na/K ratio and SBP, and illustrated the slope of the association between potassium excretion and SBP using the largest Japanese general population for the first time.

Although many guidelines, including the JSH 2019, recommend smoking cessation [1–3], the association between smoking and BP remains unclear. Additionally, smoking induces elevated BP in the short term by increasing sympathetic nerve activity, oxidative stress, and vasoconstriction [46, 47]. However, the results of previous studies on the association between smoking and BP have been inconsistent. For example, some studies have shown a positive association between smoking and BP [8–12], while others have demonstrated that smokers have lower or no significant difference in BP compared to never smokers [13–16]. Our large-scale cross-sectional study showed that current smokers who smoked 1–10 cigarettes per day and 11–20 cigarettes per day had lower SBP than never-smokers for both men and women. Given the cross-sectional nature of this study, occurrence of reverse causality is a possibility. We are currently conducting a follow-up survey; thus, we plan to conduct a prospective cohort study in a large Japanese population to clarify the relationship between smoking and the incidence of hypertension.

GGT level is known to be associated not only with alcohol consumption but also with obesity and related conditions, such as fatty liver or nonalcoholic fatty liver disease [25, 26, 48]. Although previous studies have shown that GGT level can predict hypertension independent of BMI and drinking status [27–35], a few studies have examined whether GGT level is associated with hypertension regardless of BMI or drinking status in the Japanese population [27, 33, 34]. For example, a 10-year prospective cohort study involving 77 male Japanese drinkers, GGT levels were positively associated with the development of hypertension, regardless of alcohol consumption [27]. In a cross-sectional study of 754 Japanese males, GGT levels were positively associated with BP [33]. Furthermore, a study that involved 1514 Japanese men and women demonstrated that GGT levels at baseline were not significantly associated with hypertension incidence; however, an increased change in GGT level over 3 years was associated with the incidence of hypertension, independent of BMI and drinking status [34]. Our study showed that GGT levels were positively and linearly associated with SBP in both men and women, independent of BMI and drinking status, which is consistent with previous findings and extends those findings [27, 33, 34]. Although the mechanisms underlying the association between GGT level and hypertension have not been fully elucidated, inflammation and oxidative stress have been considered as factors explaining this relationship. GGT level is positively associated with inflammation and oxidative stress markers, which may be directly related to the pathogenesis of hypertension [29]. In contrast, a Mendelian randomization study showed no positive association between GGT levels and BP [49]. Therefore, further studies are warranted to elucidate whether a causal relationship exists between GGT level and BP and to clarify the underlying mechanisms of this relationship. However, of note, participants with higher GGT levels may have elevated BP, regardless of BMI and drinking status. Therefore, lifestyle management using GGT level as a marker may be beneficial in addition to weight management and alcohol consumption.

In this study, leisure-time physical activity was not associated with SBP. Many previous studies have demonstrated that leisure-time physical activity is inversely associated with hypertension [50–52], which is inconsistent with our findings. These inconsistent results may have resulted from the initiation of exercise by participants who tended to have a higher BP. However, we could not make causal inferences because this was a cross-sectional study. Additionally, leisure-time physical activity was self-reported; thus, measurement errors may have influenced this association. The TMM CommCohort study measured objective physical activity using wearable devices and home BP, such as a pedometer in the baseline survey and a physical activity monitoring device in the detailed survey during the second period as an add-on study. Therefore, in the future, we intend to examine not only the relationship between objectively measured physical activity and future hypertension but also whether self-reported physical activity or objectively measured physical activity is strongly associated with BP.

The strength of this study is that we described in detail the association between the risk factors for hypertension (e.g., age, BMI, salt intake and Na/K ratio, each increment of 1) and SBP, as this study enrolled the largest population of approximately 62,000 Japanese. Furthermore, this study illustrated slope of the association between potassium excretion and SBP among Japanese population for the first time. Finally, to the best our knowledge, this is the first study to show an association between GGT level and SBP in a Japanese population by sex. However, our study has some limitations. First, this study was cross-sectional and could not definitively establish a causal relationship. Although previous studies have reported the direction of the relationships and/or causality between risk factors for hypertension and SBP, the association between smoking and BP remains unclear. Therefore, further prospective cohort studies are warranted to determine the effects of smoking on BP. Additionally, physical activity was evaluated using a self-report questionnaire, which may have caused measurement errors. The TMM CommCohort collected information on physical activity using a wearable device. Therefore, we examined the association between objective measurements of physical activity and BP. Second, we did not consider other risk factors for hypertension, such as sleep apnea syndrome or sleep quality [53, 54]. Third, because this study used BP measured at municipal health checkup sites, we could not show the relationship between hypertension risk factors and home BP, which can predict future cardiovascular disease better than BP measured at the office [55, 56].

### Perspective of Asia

Our findings are in accordance with the 2019 JSH guideline [1]. We further showed that GGT is positively associated with SBP regardless of hypertension risk factors including BMI and alcohol consumption, thus, lifestyle management focusing GGT levels as a marker may be also beneficial in preventing hypertension. Furthermore, we visualized relationship of Na, K, and Na/K ratio with SBP. We reconfirm the importance of salt reduction and increment of fruit/vegetable intake. Recently, a Na/K ratio self-monitoring device using spot urine was developed and its change is associated with changes in SBP [42, 45, 54, 57]. Therefore, guidance on salt reduction and potassium intake using a urinary Na/K ratio as an indicator may be useful for managing BP not only in Japan but also in Asia.

## Conclusion

SBP is associated with the risk factors for hypertension, as described in JSH 2019. Additionally, GGT levels were positively and linearly associated with SBP regardless of BMI and drinking status. Our findings support the notion that lifestyle modifications are important for preventing hypertension. Furthermore, participants with higher GGT levels may have high BP, regardless of BMI and drinking status.

### Supplementary information

Supplemental Table 1

Supplemental Table 2

Supplemental Figure 1

Supplemental Figure 2

Supplemental Figure 3

Supplemental Figure 4

Supplemental Figure 5

Supplemental Figure 6

Supplemental Figure 7

Supplemental Figure 8

Supplemental Figure 9

Supplemental Figure 10

Supplemental Figure 11

Supplemental Figure 12

Supplemental Figure Legend
