# Supplementary material for: Relationship between traditional risk factors for hypertension and systolic blood pressure in the Tohoku Medical Megabank Community-based Cohort Study
Source: Hypertens Res. 2024 Feb 29;47(6):1533–45. doi: 10.1038/s41440-024-01582-1 (PMC11150157; doi:10.1038/s41440-024-01582-1)
Supplement: Supplementary file 1 — Supplemental Table 1 [file 41440_2024_1582_MOESM1_ESM.docx]

Supplemental Table 1. Characteristics of participants

|  | Men | Women | All participants |
| --- | --- | --- | --- |
| Number | 23,446 | 38,921 | 62,367 |
| Age, years | 62.5 (10.3) | 59.6 (11.3) | 60.7 (11.0) |
| BMI, kg/m^2^ | 24.1 (3.2) | 23.1 (3.7) | 23.5 (3.5) |
| SBP, mmHg | 128.9 (16.7) | 124.7 (17.5) | 126.3 (17.3) |
| DBP, mmHg | 78.3 (10.1) | 73.8 (10.3) | 75.5 (10.4) |
| Treatment for hypertension, % | 7689 (32.8) | 9064 (23.3) | 16753 (26.9) |
| Estimated 24-h sodium excretion, mEq/day | 169.9 (39.1) | 165.2 (38.5) | 167.0 (38.8) |
| Estimated 24-h potassium excretion, mEq/day | 41.9 (9.3) | 42.0 (9.7) | 42.0 (9.5) |
| Estimated daily salt intake, g | 9.5 (2.3) | 9.2 (2.2) | 9.3 (2.3) |
| Sodium to potassium ratio | 4.2 (1.0) | 4.0 (1.0) | 4.1 (1.0) |
| Gamma-glutamyl transferase | 32.0 [22.0, 55.0] | 18.0 [14.0, 26.0] | 22.0 [16.0–36.0] |
| Physical activity, MET-min/week | 0.0 [0.0, 67.5] | 9.0 [0.0, 96.4] | 9.0 [0.0, 96.4) |
| Drinking status, % |  |  |  |
| Never-drinker | 4,967 (21.2) | 24,672 (63.4) | 29,639 (47.5) |
| Ex-drinker | 1,120 (4.8) | 663 (1.7) | 1,783 (2.9) |
| <23g/day | 7,536 (32.1) | 11,203 (28.8) | 18,715 (30.0) |
| 23-45.9g/day | 4,928 (21.0) | 1,390 (3.6) | 6,328 (10.2) |
| ≥46g/day | 4,862 (20.7) | 639 (1.6) | 5,515 (8.8) |
| Unknown | 33 (0.1) | 354 (0.9) | 387 (0.6) |
| Smoking status, % |  |  |  |
| Never-smoker | 6,043 (25.8) | 32,212 (82.8) | 38,255 (61,3) |
| Ex-smoker | 11,069 (47.2) | 3,355 (8.6) | 14,424 (23.1) |
| 1-9 cigarettes per day | 1,365 (5.8) | 1,420 (3.7) | 2,785 (4.5) |
| 10-19 cigarettes per day | 3,900 (16.6) | 955 (2.5) | 4,855 (7.8) |
| ≥20 cigarettes per day | 972 (4.2) | 86 (0.2) | 1,058 (1.7) |
| Unknown | 97 (0.4) | 893 (2.3) | 990 (1.6) |
| Education status, % |  |  |  |
| below high school | 17,021 (72.6) | 25,983 (66.8) | 43,004 (69.0) |
| vocational school, junior college, or technical college | 2,886 (12.3) | 10,367 (26.6) | 13,253 (21.3) |
| University or graduate school | 3,023 (12.9) | 1,807 (4.6) | 4,830 (7.7) |
| Unknown | 516 (2.2) | 764 (2.0) | 1,280 (2.1) |
| House damage of GEJE, % |  |  |  |
| Completely destroyed | 2,088 (8.9) | 3,636 (9.3) | 5,724 (9.2) |
| Large-scale partial collapse | 8,062 (34.4) | 13,714 (35.2) | 21,776 (34.9) |
| Partially destroyed | 7,893 (33.7) | 12,526 (32.2) | 20,419 (32.7) |
| Partially damaged | 1,277 (5.5) | 2,031 (5.2) | 3,308 (5.3) |
| No damage | 786 (3.4) | 1,295 (3.3) | 2,081 (3.3) |
| Do not live in the affected area | 2,140 (9.1) | 3,740 (9.6) | 5,880 (9.4) |
| Unknown | 1,200 (5.1) | 1,979 (5.1) | 3,179 (5.1) |
| Residential area |  |  |  |
| Miyagi | 14,029 (59.8) | 22,828 (58.7) | 36,857 (59.1) |
| Iwate | 9,417 (40.2) | 16,093 (41.4) | 25,510 (40.9) |

BMI, body mass index; DBP, diastolic blood pressure; GEJE, Great East Japan Earthquake; METs, metabolic equivalents; SBP, systolic blood pressure.

Values are expressed as mean (standard deviation) or median (interquartile range) for continuous variables or as numbers (percentages) for categorical variables.
