# Supplementary material for: Relationship between traditional risk factors for hypertension and systolic blood pressure in the Tohoku Medical Megabank Community-based Cohort Study
Source: Hypertens Res. 2024 Feb 29;47(6):1533–45. doi: 10.1038/s41440-024-01582-1 (PMC11150157; doi:10.1038/s41440-024-01582-1)
Supplement: Supplementary file 2 — Supplemental Table 2 [file 41440_2024_1582_MOESM2_ESM.docx]

Supplemental Table 2. Association between hypertension risk factors and SBP according to presence/absence obesity

| Men | | | | | | | Women | | | | | |
| --- | --- | --- | --- | --- | --- | --- | --- | --- | --- | --- | --- | --- |
| Non-obesity | | | | | | | Non-obesity | | | | | |
| Age, years | LS means of  SBP (mmHg) | 95%CI | | | P for different | P for linear trend | Age, years | LS means of SBP (mmHg) | 95%CI | | P for different | P for linear trend |
| <30 | 120.6004 | 117.6562 | 123.5446 | Ref | | <0.001 | <30 | 113.91874 | 111.67179 | 116.16569 | Ref | <0.001 |
| 31 | 114.5499 | 109.3700 | 119.7298 | 0.4012 | |  | 31 | 113.19490 | 110.14704 | 116.24276 | 1.00000 |  |
| 32 | 117.6881 | 112.6623 | 122.7139 | 0.9991 | |  | 32 | 114.35826 | 111.32149 | 117.39503 | 1.00000 |  |
| 33 | 117.2609 | 112.4465 | 122.0754 | 0.9862 | |  | 33 | 114.61235 | 111.63109 | 117.59361 | 1.00000 |  |
| 34 | 117.9889 | 113.7231 | 122.2546 | 0.9979 | |  | 34 | 115.47034 | 112.89379 | 118.04689 | 0.98440 |  |
| 35 | 118.6295 | 114.3459 | 122.9131 | 1.0000 | |  | 35 | 114.26298 | 111.63352 | 116.89245 | 1.00000 |  |
| 36 | 118.0097 | 113.6568 | 122.3626 | 0.9988 | |  | 36 | 115.98809 | 113.36886 | 118.60732 | 0.79740 |  |
| 37 | 118.3836 | 114.1207 | 122.6465 | 0.9999 | |  | 37 | 116.76382 | 114.06810 | 119.45954 | 0.34670 |  |
| 38 | 117.9184 | 114.0322 | 121.8046 | 0.9888 | |  | 38 | 116.86864 | 114.33303 | 119.40424 | 0.19120 |  |
| 39 | 121.9258 | 118.0361 | 125.8156 | 1.0000 | |  | 39 | 116.16244 | 113.57378 | 118.75111 | 0.67580 |  |
| 40 | 120.7512 | 117.1463 | 124.3562 | 1.0000 | |  | 40 | 117.74420 | 115.28068 | 120.20772 | 0.01260 |  |
| 41 | 119.0040 | 115.1753 | 122.8327 | 1.0000 | |  | 41 | 117.20877 | 114.63832 | 119.77921 | 0.10160 |  |
| 42 | 118.6362 | 114.6563 | 122.6160 | 1.0000 | |  | 42 | 118.00340 | 115.43047 | 120.57633 | 0.01120 |  |
| 43 | 118.8563 | 115.2893 | 122.4233 | 1.0000 | |  | 43 | 118.19089 | 115.63508 | 120.74669 | 0.00600 |  |
| 44 | 121.6196 | 117.9335 | 125.3057 | 1.0000 | |  | 44 | 118.41380 | 115.79674 | 121.03087 | 0.00440 |  |
| 45 | 121.0751 | 117.4710 | 124.6791 | 1.0000 | |  | 45 | 120.09431 | 117.54411 | 122.64451 | <.0001 |  |
| 46 | 122.1625 | 118.8275 | 125.4975 | 1.0000 | |  | 46 | 120.93194 | 118.37160 | 123.49228 | <.0001 |  |
| 47 | 120.0058 | 116.5263 | 123.4853 | 1.0000 | |  | 47 | 121.55983 | 118.99598 | 124.12368 | <.0001 |  |
| 48 | 122.1698 | 118.6683 | 125.6713 | 1.0000 | |  | 48 | 123.04756 | 120.46375 | 125.63137 | <.0001 |  |
| 49 | 121.4714 | 117.8738 | 125.0690 | 1.0000 | |  | 49 | 122.71918 | 120.15340 | 125.28496 | <.0001 |  |
| 50 | 121.9597 | 118.5498 | 125.3696 | 1.0000 | |  | 50 | 122.50696 | 120.05631 | 124.95761 | <.0001 |  |
| 51 | 122.5973 | 119.1877 | 126.0069 | 0.9993 | |  | 51 | 124.17842 | 121.77512 | 126.58171 | <.0001 |  |
| 52 | 124.2472 | 120.6865 | 127.8080 | 0.6670 | |  | 52 | 123.89326 | 121.52252 | 126.26400 | <.0001 |  |
| 53 | 125.2759 | 122.2191 | 128.3327 | 0.1096 | |  | 53 | 126.32975 | 124.01825 | 128.64125 | <.0001 |  |
| 54 | 123.8341 | 120.8044 | 126.8638 | 0.6153 | |  | 54 | 126.41489 | 124.10115 | 128.72862 | <.0001 |  |
| 55 | 123.4049 | 120.3559 | 126.4539 | 0.8352 | |  | 55 | 126.75820 | 124.53586 | 128.98054 | <.0001 |  |
| 56 | 124.0306 | 121.0647 | 126.9965 | 0.4947 | |  | 56 | 125.58916 | 123.31636 | 127.86195 | <.0001 |  |
| 57 | 127.0349 | 124.0695 | 130.0003 | 0.0018 | |  | 57 | 125.96391 | 123.75399 | 128.17382 | <.0001 |  |
| 58 | 127.3761 | 124.5731 | 130.1790 | 0.0004 | |  | 58 | 126.65272 | 124.48353 | 128.82192 | <.0001 |  |
| 59 | 125.1399 | 122.4135 | 127.8663 | 0.0580 | |  | 59 | 128.33240 | 126.17054 | 130.49426 | <.0001 |  |
| 60 | 127.8563 | 125.2358 | 130.4767 | <.0001 | |  | 60 | 127.37212 | 125.29063 | 129.45361 | <.0001 |  |
| 61 | 125.1662 | 122.6598 | 127.6727 | 0.0247 | |  | 61 | 128.15655 | 126.10381 | 130.20928 | <.0001 |  |
| 62 | 126.9671 | 124.5962 | 129.3381 | <.0001 | |  | 62 | 128.20341 | 126.17678 | 130.23005 | <.0001 |  |
| 63 | 127.1933 | 124.8857 | 129.5009 | <.0001 | |  | 63 | 128.13348 | 126.11056 | 130.15639 | <.0001 |  |
| 64 | 126.6329 | 124.3709 | 128.8949 | <.0001 | |  | 64 | 128.70324 | 126.70528 | 130.70120 | <.0001 |  |
| 65 | 127.0449 | 124.7823 | 129.3074 | <.0001 | |  | 65 | 128.69842 | 126.71739 | 130.67945 | <.0001 |  |
| 66 | 127.6858 | 125.4631 | 129.9085 | <.0001 | |  | 66 | 130.01906 | 128.04055 | 131.99756 | <.0001 |  |
| 67 | 128.7118 | 126.4851 | 130.9385 | <.0001 | |  | 67 | 130.60378 | 128.59599 | 132.61158 | <.0001 |  |
| 68 | 128.9022 | 126.6258 | 131.1787 | <.0001 | |  | 68 | 130.92406 | 128.86555 | 132.98258 | <.0001 |  |
| 69 | 128.3198 | 126.0392 | 130.6003 | <.0001 | |  | 69 | 131.20674 | 129.10675 | 133.30674 | <.0001 |  |
| 70 | 128.4189 | 126.1632 | 130.6746 | <.0001 | |  | 70 | 130.76271 | 128.72232 | 132.80309 | <.0001 |  |
| 71 | 128.8930 | 126.6484 | 131.1375 | <.0001 | |  | 71 | 132.06945 | 130.00814 | 134.13075 | <.0001 |  |
| 72 | 129.0991 | 126.8635 | 131.3347 | <.0001 | |  | 72 | 131.59767 | 129.53656 | 133.65878 | <.0001 |  |
| 73 | 129.6018 | 127.3371 | 131.8666 | <.0001 | |  | 73 | 132.15929 | 130.08138 | 134.23720 | <.0001 |  |
| 74 | 130.9870 | 128.6744 | 133.2995 | <.0001 | |  | 74 | 132.71992 | 130.60073 | 134.83911 | <.0001 |  |
| ≥75 | 130.5014 | 126.1182 | 134.8847 | 0.0007 | |  | ≥75 | 136.67338 | 132.31712 | 141.02965 | <.0001 |  |
| BMI, kg/m^2^ |  |  |  |  | |  | BMI, kg/m^2^ |  |  |  |  |  |
| <19.0 | 120.7895 | 118.5029 | 123.0760 | Ref | | <0.001 | <19.0 | 119.8350 | 117.9451 | 121.7250 | Ref | <0.001 |
| 19.0-19.9 | 121.4846 | 119.2409 | 123.7284 | 0.7716 | |  | 19.0-19.9 | 121.7578 | 119.8469 | 123.6686 | <.0001 |  |
| 20.0-20.9 | 123.7940 | 121.6543 | 125.9336 | <.0001 | |  | 20.0-20.9 | 122.3620 | 120.4755 | 124.2484 | <.0001 |  |
| 21.0-21.9 | 123.6366 | 121.5381 | 125.7351 | <.0001 | |  | 21.0-21.9 | 124.0079 | 122.1260 | 125.8899 | <.0001 |  |
| 22.0-22.9 | 124.7316 | 122.6546 | 126.8086 | <.0001 | |  | 22.0-22.9 | 124.8069 | 122.9220 | 126.6919 | <.0001 |  |
| 23.0-23.9 | 125.2958 | 123.2279 | 127.3636 | <.0001 | |  | 23.0-23.9 | 126.3876 | 124.5010 | 128.2741 | <.0001 |  |
| 24.0-24.9 | 125.4663 | 123.3925 | 127.5402 | <.0001 | |  | 24.0-24.9 | 127.3420 | 125.4542 | 129.2297 | <.0001 |  |
| Smoking status |  |  |  |  | |  | Smoking status |  |  |  |  |  |
| Never smoker | 124.6555 | 122.6449 | 126.6660 | Ref | |  | Never smoker | 124.7016 | 123.0062 | 126.3969 | Ref |  |
| Current smoker (1-10 cigarettes/day) | 123.0012 | 120.8279 | 125.1745 | 0.0209 | |  | Current smoker (1-10 cigarettes/day) | 123.3624 | 121.4727 | 125.2521 | 0.0358 |  |
| Current smoker (11-20 cigarettes/day) | 123.2280 | 121.2021 | 125.2540 | 0.0032 | |  | Current smoker (11-20 cigarettes/day) | 122.8068 | 120.7973 | 124.8164 | 0.0107 |  |
| Current smoker (≥21 cigarettes/day) | 123.0624 | 120.7355 | 125.3893 | 0.1070 | |  | Current smoker (≥21 cigarettes/day) | 122.8849 | 118.1295 | 127.6402 | 0.9374 |  |
| Ex-smoker | 123.9840 | 121.9971 | 125.9710 | 0.1834 | |  | Ex-smoker | 123.6649 | 121.8873 | 125.4426 | 0.0137 |  |
| Unknown | 123.6674 | 119.4002 | 127.9346 | 0.9908 | |  | Unknown | 125.2930 | 123.1838 | 127.4022 | 0.9282 |  |
| Drinking status |  |  |  |  | |  | Drinking status |  |  |  |  |  |
| Never drinker | 122.6094 | 120.7440 | 124.4747 | Ref | |  | Never drinker | 122.4502 | 120.6152 | 124.2852 | Ref |  |
| Current drinker (<23g/day) | 123.4721 | 121.6371 | 125.3071 | 0.0906 | |  | Current drinker (<23g/day) | 122.2016 | 120.3655 | 124.0378 | 0.7544 |  |
| Current drinker(23-45g) | 126.1957 | 124.3457 | 128.0457 | <.0001 | |  | Current drinker(23-45g) | 125.3136 | 123.3147 | 127.3125 | <.0001 |  |
| Current drinker (≥46g/day) | 128.2771 | 126.4556 | 130.0986 | <.0001 | |  | Current drinker (≥46g/day) | 127.1364 | 124.9560 | 129.3169 | <.0001 |  |
| Ex-drinker | 120.5199 | 118.3751 | 122.6646 | 0.0095 | |  | Ex-drinker | 120.8061 | 118.5323 | 123.0799 | 0.1207 |  |
| Unknown | 120.5245 | 114.1367 | 126.9123 | 0.9600 | |  | Unknown | 124.8056 | 121.9773 | 127.6340 | 0.1893 |  |
| Estimated daily salt intake, g/day |  |  |  |  | |  | Estimated daily salt intake, g/day |  |  |  |  |  |
| <6.0 | 116.8532 | 114.5667 | 119.1397 | Ref | | <0.001 | <6.0 | 116.3482 | 114.5253 | 118.1712 | Ref | <0.001 |
| 6.0-6.9 | 120.1804 | 118.0754 | 122.2854 | 0.0012 | |  | 6.0-6.9 | 117.2907 | 115.5493 | 119.0321 | 0.5478 |  |
| 7.0-7.9 | 120.1855 | 118.2100 | 122.1610 | 0.0002 | |  | 7.0-7.9 | 118.4379 | 116.7468 | 120.1291 | 0.0006 |  |
| 8.0-8.9 | 120.9719 | 119.0059 | 122.9380 | <.0001 | |  | 8.0-8.9 | 119.1333 | 117.4541 | 120.8124 | <.0001 |  |
| 9.0-9.9 | 122.3547 | 120.4049 | 124.3045 | <.0001 | |  | 9.0-9.9 | 119.6487 | 117.9758 | 121.3216 | <.0001 |  |
| 10.0-10.9 | 122.7751 | 120.8179 | 124.7324 | <.0001 | |  | 10.0-10.9 | 120.8640 | 119.1783 | 122.5497 | <.0001 |  |
| 11.0-11.9 | 123.9840 | 121.9817 | 125.9863 | <.0001 | |  | 11.0-11.9 | 122.1497 | 120.4404 | 123.8591 | <.0001 |  |
| 12.0-12.9 | 125.1200 | 123.0381 | 127.2020 | <.0001 | |  | 12.0-12.9 | 123.0679 | 121.2959 | 124.8400 | <.0001 |  |
| 13.0-13.9 | 125.1339 | 122.8729 | 127.3949 | <.0001 | |  | 13.0-13.9 | 124.8683 | 122.9559 | 126.7807 | <.0001 |  |
| 14.0-14.9 | 128.9940 | 126.3157 | 131.6722 | <.0001 | |  | 14.0-14.9 | 125.2083 | 122.9653 | 127.4513 | <.0001 |  |
| 15.0-15.9 | 127.8317 | 124.4750 | 131.1884 | <.0001 | |  | 15.0-15.9 | 127.2030 | 124.2706 | 130.1354 | <.0001 |  |
| 16.0-16.9 | 128.2307 | 123.6066 | 132.8548 | <.0001 | |  | 16.0-16.9 | 128.8407 | 124.5042 | 133.1773 | <.0001 |  |
| 17.0-17.9 | 124.7192 | 118.5038 | 130.9346 | 0.0979 | |  | 17.0-17.9 | 136.7771 | 128.8091 | 144.7450 | <.0001 |  |
| ≥18.0 | 123.0623 | 115.4391 | 130.6855 | 0.5846 | |  | ≥18.0 | 133.1605 | 124.9387 | 141.3822 | 0.0006 |  |
| Estimated potassium excretion, mEq/day |  |  |  |  | |  | Estimated potassium excretion, mEq/day |  |  |  |  |  |
| <25.0 | 124.3336 | 121.5762 | 127.0910 | Ref | | <0.001 | <25.0 | 126.3641 | 124.1427 | 128.5856 | Ref | <0.001 |
| 25.0-29.9 | 126.4467 | 124.2616 | 128.6318 | 0.1660 | |  | 25.0-29.9 | 126.0439 | 124.1043 | 127.9834 | 0.9943 |  |
| 30.0-34.9 | 126.1332 | 124.0283 | 128.2382 | 0.2625 | |  | 30.0-34.9 | 125.4076 | 123.5126 | 127.3025 | 0.4630 |  |
| 35.0-39.9 | 125.5359 | 123.4464 | 127.6255 | 0.6252 | |  | 35.0-39.9 | 124.9837 | 123.0973 | 126.8700 | 0.1527 |  |
| 40.0-44.9 | 124.1665 | 122.0788 | 126.2542 | 1.0000 | |  | 40.0-44.9 | 124.7185 | 122.8394 | 126.5976 | 0.0660 |  |
| 45.0-49.9 | 123.4387 | 121.3344 | 125.5430 | 0.8483 | |  | 45.0-49.9 | 123.0500 | 121.1644 | 124.9356 | <.0001 |  |
| 50.0-54.9 | 123.4793 | 121.3317 | 125.6269 | 0.8870 | |  | 50.0-54.9 | 122.8852 | 120.9676 | 124.8029 | <.0001 |  |
| 55.0-59.9 | 122.7911 | 120.5167 | 125.0656 | 0.5190 | |  | 55.0-59.9 | 121.3416 | 119.3591 | 123.3242 | <.0001 |  |
| 60.0-64.9 | 121.0877 | 118.3840 | 123.7914 | 0.0806 | |  | 60.0-64.9 | 122.4487 | 120.2451 | 124.6524 | 0.0002 |  |
| ≥65.0 | 118.5849 | 115.3112 | 121.8586 | 0.0040 | |  | ≥65.0 | 120.6127 | 118.2290 | 122.9964 | <.0001 |  |
| GGT, IU/L |  |  |  |  | |  | GGT, IU/L |  |  |  |  |  |
| <25.0 | 120.4967 | 118.4694 | 122.5239 | Ref | | <0.001 | <25.0 | 121.9394 | 120.1617 | 123.7172 | Ref | <0.001 |
| 25.0-49.9 | 122.5951 | 120.5604 | 124.6298 | <.0001 | |  | 25.0-49.9 | 123.9050 | 122.0949 | 125.7151 | <.0001 |  |
| 50.0-74.9 | 123.7217 | 121.5872 | 125.8562 | <.0001 | |  | 50.0-74.9 | 123.9043 | 121.8654 | 125.9431 | 0.0010 |  |
| 75.0-99.9 | 124.9257 | 122.6309 | 127.2205 | <.0001 | |  | 75.0-99.9 | 123.7376 | 121.2723 | 126.2029 | 0.1603 |  |
| ≥100 | 126.2597 | 124.0796 | 128.4397 | <.0001 | |  | ≥100 | 125.4417 | 123.0623 | 127.8211 | 0.0001 |  |
| Leisure-time physical activity, METs-min/week |  |  |  |  | |  | Leisure-time physical activity, METs-min/week |  |  |  |  |  |
| <50.0 | 123.1653 | 121.5946 | 124.7360 | Ref | | 0.7120 | <50.0 | 124.2318 | 122.8332 | 125.6303 | Ref | 0.193 |
| 50.0-99.9 | 123.3016 | 121.5268 | 125.0764 | 1.0000 | |  | 50.0-99.9 | 124.1624 | 122.6556 | 125.6693 | 1.0000 |  |
| 100.0-149.9 | 122.9934 | 121.1401 | 124.8467 | 1.0000 | |  | 100.0-149.9 | 124.1312 | 122.5994 | 125.6630 | 1.0000 |  |
| 150.0-199.9 | 122.1161 | 119.7503 | 124.4820 | 0.9970 | |  | 150.0-199.9 | 124.0953 | 122.1252 | 126.0654 | 1.0000 |  |
| 200.0-249.9 | 123.6279 | 121.4155 | 125.8402 | 1.0000 | |  | 200.0-249.9 | 124.6464 | 122.8520 | 126.4408 | 1.0000 |  |
| 250.0-299.9 | 122.9996 | 120.8485 | 125.1506 | 1.0000 | |  | 250.0-299.9 | 124.1865 | 122.4799 | 125.8932 | 1.0000 |  |
| 300.0-349.9 | 122.2821 | 119.6536 | 124.9105 | 1.0000 | |  | 300.0-349.9 | 123.3858 | 121.2975 | 125.4742 | 0.9990 |  |
| 350.0-399.9 | 122.6571 | 119.4965 | 125.8177 | 1.0000 | |  | 350.0-399.9 | 121.1132 | 118.3510 | 123.8754 | 0.1946 |  |
| 400.0-449.9 | 124.3384 | 119.6326 | 129.0441 | 1.0000 | |  | 400.0-449.9 | 122.3431 | 118.4303 | 126.2558 | 0.9994 |  |
| 450.0-499.9 | 123.1892 | 120.8213 | 125.5572 | 1.0000 | |  | 450.0-499.9 | 124.3943 | 122.5643 | 126.2243 | 1.0000 |  |
| 500.0-549.9 | 124.1831 | 119.6456 | 128.7207 | 1.0000 | |  | 500.0-549.9 | 128.9865 | 124.9543 | 133.0186 | 0.2436 |  |
| 550.0-599.9 | 121.4465 | 114.9850 | 127.9080 | 1.0000 | |  | 550.0-599.9 | 125.5782 | 120.4586 | 130.6979 | 1.0000 |  |
| 600.0-649.9 | 122.1702 | 119.1334 | 125.2070 | 1.0000 | |  | 600.0-649.9 | 123.5457 | 121.2993 | 125.7921 | 1.0000 |  |
| 650.0-699.9 | 126.7811 | 118.8000 | 134.7622 | 0.9999 | |  | 650.0-699.9 | 123.7908 | 117.8309 | 129.7507 | 1.0000 |  |
| 700.0-749.9 | 124.0452 | 121.3660 | 126.7243 | 1.0000 | |  | 700.0-749.9 | 122.9233 | 120.8814 | 124.9652 | 0.8565 |  |
| 750.0-799.9 | 132.0653 | 123.8364 | 140.2942 | 0.4662 | |  | 750.0-799.9 | 128.1297 | 119.9625 | 136.2969 | 0.9998 |  |
| 800.0-849.9 | 120.0918 | 110.9196 | 129.2640 | 1.0000 | |  | 800.0-849.9 | 127.0801 | 120.2903 | 133.8699 | 1.0000 |  |
| 850.0-899.9 | 124.6126 | 113.4328 | 135.7925 | 1.0000 | |  | 850.0-899.9 | 117.2068 | 101.5613 | 132.8522 | 0.9999 |  |
| 900.0-949.9 | 122.3279 | 113.8040 | 130.8518 | 1.0000 | |  | 900.0-949.9 | 123.5219 | 117.3561 | 129.6876 | 1.0000 |  |
| 950.0-999.9 | 123.0715 | 110.2002 | 135.9428 | 1.0000 | |  | 950.0-999.9 | 118.6220 | 109.1180 | 128.1260 | 0.9960 |  |
| ≥1000.0 | 124.1292 | 119.0116 | 129.2468 | 1.0000 | |  | ≥1000.0 | 123.4225 | 117.2661 | 129.5788 | 1.0000 |  |
| Urinary sodium-to-potassium ratio |  |  |  |  | |  | Urinary sodium-to-potassium ratio |  |  |  |  |  |
| ＜3.0 | 118.4971 | 114.7010 | 122.2932 | Ref | | <0.001 | ＜3.0 | 114.6766 | 111.8250 | 117.5283 | Ref | <0.001 |
| 3.0-3.9 | 120.0687 | 118.0946 | 122.0428 | 0.7321 | |  | 3.0-3.9 | 117.8240 | 116.1466 | 119.5014 | 0.0391 |  |
| 4.0-4.9 | 121.8024 | 119.9295 | 123.6752 | 0.1539 | |  | 4.0-4.9 | 119.8188 | 118.1943 | 121.4433 | 0.0001 |  |
| 5.0-5.9 | 123.9864 | 122.1039 | 125.8690 | 0.0052 | |  | 5.0-5.9 | 121.5951 | 119.9613 | 123.2289 | <.0001 |  |
| 6.0-6.9 | 125.4079 | 123.4492 | 127.3666 | 0.0003 | |  | 6.0-6.9 | 122.8434 | 121.1536 | 124.5333 | <.0001 |  |
| 7.0-7.9 | 127.0979 | 124.8093 | 129.3864 | <.0001 | |  | 7.0-7.9 | 124.9782 | 123.0051 | 126.9512 | <.0001 |  |
| 8.0-8.9 | 125.0684 | 121.8780 | 128.2588 | 0.0089 | |  | 8.0-8.9 | 125.1960 | 121.9930 | 128.3991 | <.0001 |  |
| ≥9.0 | 126.6032 | 120.9818 | 132.2245 | 0.0365 | |  | ≥9.0 | 124.3745 | 118.5334 | 130.2155 | 0.0071 |  |
| Obesity | | |  | | | | Obesity | | |  | | |
| Age, years |  |  |  |  | |  | Age, years |  |  |  |  |  |
| <30 | 130.0626 | 125.6576 | 134.4676 | Ref | | <0.001 | <30 | 121.0210 | 116.8769 | 125.1651 | Ref | <0.001 |
| 31 | 126.9400 | 118.6052 | 135.2749 | 1.0000 | |  | 31.0000 | 127.4708 | 120.4699 | 134.4717 | 0.7214 |  |
| 32 | 133.5882 | 127.1736 | 140.0029 | 0.9992 | |  | 32.0000 | 119.3623 | 112.6582 | 126.0663 | 1.0000 |  |
| 33 | 125.7702 | 118.7449 | 132.7954 | 0.9949 | |  | 33.0000 | 122.2232 | 116.5809 | 127.8655 | 1.0000 |  |
| 34 | 131.4842 | 125.1022 | 137.8662 | 1.0000 | |  | 34.0000 | 122.2858 | 116.9513 | 127.6202 | 1.0000 |  |
| 35 | 128.6242 | 122.7149 | 134.5335 | 1.0000 | |  | 35.0000 | 124.4616 | 119.6157 | 129.3074 | 0.9669 |  |
| 36 | 131.3629 | 124.8241 | 137.9018 | 1.0000 | |  | 36.0000 | 121.1145 | 115.8502 | 126.3789 | 1.0000 |  |
| 37 | 127.1223 | 121.3321 | 132.9126 | 0.9998 | |  | 37.0000 | 124.1567 | 119.3046 | 129.0088 | 0.9888 |  |
| 38 | 128.8887 | 123.8738 | 133.9037 | 1.0000 | |  | 38.0000 | 126.8791 | 122.1170 | 131.6411 | 0.3160 |  |
| 39 | 129.6501 | 123.8613 | 135.4389 | 1.0000 | |  | 39.0000 | 128.2681 | 123.2876 | 133.2486 | 0.1206 |  |
| 40 | 134.1288 | 129.2162 | 139.0414 | 0.8576 | |  | 40.0000 | 125.5227 | 121.1489 | 129.8966 | 0.6142 |  |
| 41 | 130.4171 | 125.8539 | 134.9803 | 1.0000 | |  | 41.0000 | 128.0034 | 123.6101 | 132.3966 | 0.0712 |  |
| 42 | 131.8244 | 127.0210 | 136.6278 | 1.0000 | |  | 42.0000 | 124.7365 | 120.3162 | 129.1568 | 0.8742 |  |
| 43 | 132.2257 | 127.6134 | 136.8380 | 1.0000 | |  | 43.0000 | 126.4059 | 121.8816 | 130.9302 | 0.3851 |  |
| 44 | 132.3309 | 127.4493 | 137.2125 | 1.0000 | |  | 44.0000 | 127.7469 | 123.2865 | 132.2074 | 0.1012 |  |
| 45 | 128.3948 | 123.5855 | 133.2041 | 1.0000 | |  | 45.0000 | 128.5623 | 124.3527 | 132.7718 | 0.0248 |  |
| 46 | 130.5185 | 125.6364 | 135.4006 | 1.0000 | |  | 46.0000 | 129.8942 | 125.8422 | 133.9461 | 0.0019 |  |
| 47 | 128.8258 | 124.2019 | 133.4496 | 1.0000 | |  | 47.0000 | 132.0643 | 127.8842 | 136.2444 | <.0001 |  |
| 48 | 134.1871 | 129.4665 | 138.9077 | 0.8063 | |  | 48.0000 | 130.4737 | 126.3538 | 134.5936 | 0.0008 |  |
| 49 | 132.3378 | 127.5372 | 137.1383 | 0.9999 | |  | 49.0000 | 131.9114 | 127.9246 | 135.8981 | <.0001 |  |
| 50 | 131.7031 | 127.0130 | 136.3932 | 1.0000 | |  | 50.0000 | 133.2232 | 129.3201 | 137.1264 | <.0001 |  |
| 51 | 134.7393 | 130.0226 | 139.4561 | 0.6141 | |  | 51.0000 | 131.6817 | 127.6806 | 135.6828 | <.0001 |  |
| 52 | 134.9328 | 130.6004 | 139.2653 | 0.4121 | |  | 52.0000 | 131.7755 | 127.8540 | 135.6970 | <.0001 |  |
| 53 | 134.5935 | 130.1469 | 139.0400 | 0.5783 | |  | 53.0000 | 132.3853 | 128.5768 | 136.1938 | <.0001 |  |
| 54 | 134.9398 | 130.6168 | 139.2627 | 0.4013 | |  | 54.0000 | 132.1991 | 128.4922 | 135.9059 | <.0001 |  |
| 55 | 136.7335 | 132.4338 | 141.0331 | 0.0569 | |  | 55.0000 | 131.1506 | 127.5723 | 134.7290 | <.0001 |  |
| 56 | 134.9148 | 130.7117 | 139.1179 | 0.3829 | |  | 56.0000 | 131.0872 | 127.5526 | 134.6218 | <.0001 |  |
| 57 | 135.8988 | 131.7357 | 140.0619 | 0.1248 | |  | 57.0000 | 131.8645 | 128.3487 | 135.3802 | <.0001 |  |
| 58 | 134.7637 | 130.7402 | 138.7871 | 0.3517 | |  | 58.0000 | 133.7461 | 130.3278 | 137.1643 | <.0001 |  |
| 59 | 138.2187 | 134.1381 | 142.2993 | 0.0032 | |  | 59.0000 | 131.7340 | 128.3707 | 135.0974 | <.0001 |  |
| 60 | 135.5448 | 131.7905 | 139.2991 | 0.0957 | |  | 60.0000 | 134.8287 | 131.5938 | 138.0636 | <.0001 |  |
| 61 | 135.7791 | 132.0748 | 139.4833 | 0.0596 | |  | 61.0000 | 134.2940 | 131.1007 | 137.4873 | <.0001 |  |
| 62 | 135.4504 | 131.8752 | 139.0256 | 0.0796 | |  | 62.0000 | 134.2312 | 131.0836 | 137.3789 | <.0001 |  |
| 63 | 135.2907 | 131.7817 | 138.7997 | 0.0846 | |  | 63.0000 | 133.4445 | 130.3522 | 136.5368 | <.0001 |  |
| 64 | 136.3390 | 132.8501 | 139.8279 | 0.0131 | |  | 64.0000 | 134.3662 | 131.2758 | 137.4566 | <.0001 |  |
| 65 | 136.0694 | 132.6369 | 139.5018 | 0.0179 | |  | 65.0000 | 135.5657 | 132.5286 | 138.6029 | <.0001 |  |
| 66 | 135.3771 | 131.9677 | 138.7865 | 0.0602 | |  | 66.0000 | 135.0831 | 132.0478 | 138.1185 | <.0001 |  |
| 67 | 135.1638 | 131.7329 | 138.5946 | 0.0865 | |  | 67.0000 | 133.4655 | 130.3790 | 136.5519 | <.0001 |  |
| 68 | 135.4144 | 131.9105 | 138.9182 | 0.0744 | |  | 68.0000 | 134.6861 | 131.5512 | 137.8210 | <.0001 |  |
| 69 | 137.7790 | 134.2193 | 141.3387 | 0.0008 | |  | 69.0000 | 134.9681 | 131.8103 | 138.1260 | <.0001 |  |
| 70 | 136.8315 | 133.4073 | 140.2556 | 0.0053 | |  | 70.0000 | 135.4936 | 132.3751 | 138.6122 | <.0001 |  |
| 71 | 135.9263 | 132.4701 | 139.3826 | 0.0274 | |  | 71.0000 | 135.6087 | 132.5014 | 138.7160 | <.0001 |  |
| 72 | 136.8702 | 133.3790 | 140.3614 | 0.0048 | |  | 72.0000 | 134.7708 | 131.6578 | 137.8837 | <.0001 |  |
| 73 | 136.7394 | 133.2436 | 140.2351 | 0.0071 | |  | 73.0000 | 135.5164 | 132.3735 | 138.6592 | <.0001 |  |
| 74 | 136.2606 | 132.7024 | 139.8188 | 0.0191 | |  | 74.0000 | 136.0099 | 132.8462 | 139.1736 | <.0001 |  |
| ≥75 | 137.6562 | 132.0291 | 143.2832 | 0.1729 | |  | ≥75 | 136.2801 | 129.7706 | 142.7895 | 0.0003 |  |
| BMI, kg/m^2^ |  |  |  |  | |  | BMI, kg/m^2^ |  |  |  |  |  |
| 25.0-25.9 | 129.7929 | 126.6004 | 132.9854 | Ref | | <0.001 | 25.0-25.9 | 125.8207 | 123.0243 | 128.6171 | Ref | <0.001 |
| 26.0-26.9 | 130.6369 | 127.4224 | 133.8513 | 0.5187 | |  | 26.0-26.9 | 127.1063 | 124.2649 | 129.9478 | 0.0555 |  |
| 27.0-27.9 | 130.5146 | 127.2681 | 133.7611 | 0.8300 | |  | 27.0-27.9 | 128.1943 | 125.3279 | 131.0608 | <.0001 |  |
| 28.0-28.9 | 131.8025 | 128.5128 | 135.0922 | 0.0089 | |  | 28.0-28.9 | 128.2523 | 125.3644 | 131.1401 | 0.0002 |  |
| 29.0-29.9 | 132.4950 | 129.1398 | 135.8501 | 0.0018 | |  | 29.0-29.9 | 129.7914 | 126.8426 | 132.7403 | <.0001 |  |
| 30.0-30.9 | 131.5076 | 127.9779 | 135.0374 | 0.4216 | |  | 30.0-30.9 | 130.1575 | 127.1196 | 133.1954 | <.0001 |  |
| 31.0-31.9 | 133.0874 | 129.3272 | 136.8476 | 0.0309 | |  | 31.0-31.9 | 130.0808 | 126.9529 | 133.2087 | <.0001 |  |
| 32.0-32.9 | 136.7911 | 132.4886 | 141.0937 | <.0001 | |  | 32.0-32.9 | 131.5410 | 128.2100 | 134.8721 | <.0001 |  |
| 33.0-33.9 | 136.0766 | 131.7679 | 140.3853 | 0.0006 | |  | 33.0-33.9 | 131.8490 | 128.2744 | 135.4237 | <.0001 |  |
| 34.0-34.9 | 138.3836 | 133.1692 | 143.5980 | 0.0011 | |  | 34.0-34.9 | 136.3014 | 132.4495 | 140.1532 | <.0001 |  |
| ≥35.0 | 136.8413 | 132.6923 | 140.9903 | <.0001 | |  | ≥35.0 | 136.1718 | 132.8687 | 139.4749 | <.0001 |  |
| Smoking status |  |  |  |  | |  | Smoking status |  |  |  |  |  |
| Never smoker | 134.0610 | 130.9122 | 137.2098 | Ref | |  | Never smoker | 132.6263 | 129.9739 | 135.2786 | Ref |  |
| Current smoker (1-10 cigarettes/day) | 131.7829 | 128.3402 | 135.2257 | 0.0417 | |  | Current smoker (1-10 cigarettes/day) | 129.4500 | 126.2675 | 132.6324 | 0.0070 |  |
| Current smoker (11-20 cigarettes/day) | 132.6289 | 129.4337 | 135.8242 | 0.0648 | |  | Current smoker (11-20 cigarettes/day) | 129.6427 | 126.3392 | 132.9462 | 0.0365 |  |
| Current smoker (≥21 cigarettes/day) | 132.1173 | 128.6047 | 135.6299 | 0.1700 | |  | Current smoker (≥21 cigarettes/day) | 127.9790 | 122.1612 | 133.7967 | 0.3598 |  |
| Ex-smoker | 133.4120 | 130.2950 | 136.5291 | 0.4610 | |  | Ex-smoker | 130.6791 | 127.8542 | 133.5040 | 0.0078 |  |
| Unknown | 136.6866 | 130.6341 | 142.7391 | 0.8649 | |  | Unknown | 132.4956 | 129.2549 | 135.7364 | 1.0000 |  |
| Drinking status |  |  |  |  | |  | Drinking status |  |  |  |  |  |
| Never drinker | 133.6040 | 130.9445 | 136.2636 | Ref | |  | Never drinker | 129.5577 | 126.8125 | 132.3029 | Ref |  |
| Current drinker (<23g/day) | 133.9487 | 131.3113 | 136.5861 | 0.9439 | |  | Current drinker (<23g/day) | 128.8983 | 126.1378 | 131.6587 | 0.3366 |  |
| Current drinker(23-45g) | 135.4272 | 132.7497 | 138.1048 | 0.0044 | |  | Current drinker(23-45g) | 130.3465 | 127.0958 | 133.5971 | 0.9417 |  |
| Current drinker (≥46g/day) | 137.0218 | 134.3693 | 139.6743 | <.0001 | |  | Current drinker (≥46g/day) | 135.1146 | 131.2412 | 138.9881 | 0.0014 |  |
| Ex-drinker | 132.2576 | 129.2729 | 135.2422 | 0.4129 | |  | Ex-drinker | 127.6348 | 124.0652 | 131.2045 | 0.4844 |  |
| Unknown | 128.4294 | 116.4691 | 140.3898 | 0.8900 | |  | Unknown | 131.3208 | 127.3280 | 135.3136 | 0.7796 |  |
| Estimated daily salt intake, g/day |  |  |  |  | |  | Estimated daily salt intake, g/day |  |  |  |  |  |
| <6.0 | 127.3044 | 123.6160 | 130.9927 | Ref | | <0.001 | <6.0 | 125.5242 | 122.2215 | 128.8269 | Ref | <0.001 |
| 6.0-6.9 | 129.0842 | 125.5804 | 132.5880 | 0.7559 | |  | 6.0-6.9 | 126.0921 | 123.0189 | 129.1652 | 0.9999 |  |
| 7.0-7.9 | 129.1895 | 125.9186 | 132.4605 | 0.5889 | |  | 7.0-7.9 | 126.4432 | 123.5481 | 129.3383 | 0.9785 |  |
| 8.0-8.9 | 130.4669 | 127.2369 | 133.6970 | 0.0572 | |  | 8.0-8.9 | 127.8596 | 125.0399 | 130.6793 | 0.1929 |  |
| 9.0-9.9 | 131.3376 | 128.1276 | 134.5476 | 0.0051 | |  | 9.0-9.9 | 128.0891 | 125.2786 | 130.8995 | 0.1129 |  |
| 10.0-10.9 | 131.1066 | 127.9053 | 134.3080 | 0.0105 | |  | 10.0-10.9 | 128.8692 | 126.0694 | 131.6690 | 0.0151 |  |
| 11.0-11.9 | 132.9108 | 129.6855 | 136.1361 | <.0001 | |  | 11.0-11.9 | 129.9823 | 127.1672 | 132.7975 | 0.0004 |  |
| 12.0-12.9 | 131.9888 | 128.7023 | 135.2753 | 0.0014 | |  | 12.0-12.9 | 130.7043 | 127.8250 | 133.5835 | <.0001 |  |
| 13.0-13.9 | 134.7295 | 131.3411 | 138.1178 | <.0001 | |  | 13.0-13.9 | 131.4123 | 128.4551 | 134.3695 | <.0001 |  |
| 14.0-14.9 | 135.5164 | 131.9576 | 139.0752 | <.0001 | |  | 14.0-14.9 | 132.8439 | 129.6640 | 136.0239 | <.0001 |  |
| 15.0-15.9 | 135.8505 | 131.6796 | 140.0214 | <.0001 | |  | 15.0-15.9 | 133.3223 | 129.7297 | 136.9150 | <.0001 |  |
| 16.0-16.9 | 137.5216 | 132.5901 | 142.4531 | <.0001 | |  | 16.0-16.9 | 134.9378 | 130.5524 | 139.3232 | <.0001 |  |
| 17.0-17.9 | 144.7735 | 138.3458 | 151.2012 | <.0001 | |  | 17.0-17.9 | 134.4297 | 128.5100 | 140.3495 | 0.0182 |  |
| ≥18.0 | 136.4936 | 129.5297 | 143.4575 | 0.0520 | |  | ≥18.0 | 136.1929 | 129.9438 | 142.4420 | 0.0047 |  |
| Estimated potassium excretion, mEq/day |  |  |  |  | |  | Estimated potassium excretion, mEq/day |  |  |  |  |  |
| <25.0 | 133.3441 | 128.3321 | 138.3560 | Ref | | <0.001 | <25.0 | 130.8278 | 125.7352 | 135.9204 | Ref | <0.001 |
| 25.0-29.9 | 134.9689 | 131.4912 | 138.4466 | 0.7765 | |  | 25.0-29.9 | 130.2321 | 127.1074 | 133.3567 | 0.9963 |  |
| 30.0-34.9 | 135.4090 | 132.1102 | 138.7078 | 0.5948 | |  | 30.0-34.9 | 131.6820 | 128.7980 | 134.5661 | 0.9685 |  |
| 35.0-39.9 | 134.7856 | 131.5401 | 138.0311 | 0.8178 | |  | 35.0-39.9 | 132.4650 | 129.6270 | 135.3031 | 0.7393 |  |
| 40.0-44.9 | 134.6983 | 131.4721 | 137.9245 | 0.8484 | |  | 40.0-44.9 | 132.3369 | 129.5122 | 135.1617 | 0.7832 |  |
| 45.0-49.9 | 133.9450 | 130.7019 | 137.1881 | 0.9967 | |  | 45.0-49.9 | 130.7485 | 127.9167 | 133.5803 | 1.0000 |  |
| 50.0-54.9 | 133.2431 | 129.9619 | 136.5242 | 1.0000 | |  | 50.0-54.9 | 129.7614 | 126.9161 | 132.6067 | 0.9242 |  |
| 55.0-59.9 | 130.7806 | 127.4216 | 134.1397 | 0.4613 | |  | 55.0-59.9 | 129.5287 | 126.5968 | 132.4607 | 0.8632 |  |
| 60.0-64.9 | 131.9814 | 128.3586 | 135.6042 | 0.8874 | |  | 60.0-64.9 | 128.5878 | 125.4138 | 131.7618 | 0.5847 |  |
| ≥65.0 | 131.3253 | 127.2424 | 135.4082 | 0.7353 | |  | ≥65.0 | 128.6174 | 125.3019 | 131.9329 | 0.6156 |  |
| GGT, IU/L |  |  |  |  | |  | GGT, IU/L |  |  |  |  |  |
| <25.0 | 131.6083 | 128.3593 | 134.8572 | Ref | | <0.001 | <25.0 | 129.5775 | 126.8343 | 132.3207 | Ref | 0.005 |
| 25.0-49.9 | 132.9342 | 129.7367 | 136.1317 | 0.0159 | |  | 25.0-49.9 | 131.1682 | 128.4022 | 133.9342 | <.0001 |  |
| 50.0-74.9 | 134.2632 | 131.0173 | 137.5091 | <.0001 | |  | 50.0-74.9 | 131.2595 | 128.3036 | 134.2154 | 0.0468 |  |
| 75.0-99.9 | 133.8167 | 130.4573 | 137.1760 | 0.0117 | |  | 75.0-99.9 | 130.1449 | 126.8478 | 133.4421 | 0.9644 |  |
| ≥100 | 134.6183 | 131.3166 | 137.9200 | <.0001 | |  | ≥100 | 130.2438 | 126.9237 | 133.5638 | 0.9429 |  |
| Leisure-time physical activity, METs-min/week |  |  |  |  | |  | Leisure-time physical activity, METs-min/week |  |  |  |  |  |
| <50.0 | 132.6964 | 130.2966 | 135.0962 | Ref | | 0.5680 | <50.0 | 129.5977 | 127.8842 | 131.3113 | Ref | 0.45 |
| 50.0-99.9 | 132.6682 | 129.9927 | 135.3437 | 1.0000 | |  | 50.0-99.9 | 130.0294 | 128.0368 | 132.0220 | 1.0000 |  |
| 100.0-149.9 | 132.5703 | 129.7486 | 135.3921 | 1.0000 | |  | 100.0-149.9 | 129.5807 | 127.5446 | 131.6168 | 1.0000 |  |
| 150.0-199.9 | 134.0617 | 130.5048 | 137.6186 | 0.9992 | |  | 150.0-199.9 | 128.3039 | 125.1100 | 131.4978 | 0.9998 |  |
| 200.0-249.9 | 131.2688 | 127.8975 | 134.6400 | 0.9943 | |  | 200.0-249.9 | 129.6657 | 127.1197 | 132.2116 | 1.0000 |  |
| 250.0-299.9 | 132.9967 | 129.7577 | 136.2357 | 1.0000 | |  | 250.0-299.9 | 130.0718 | 127.6854 | 132.4582 | 1.0000 |  |
| 300.0-349.9 | 129.9804 | 126.1560 | 133.8048 | 0.7756 | |  | 300.0-349.9 | 129.9564 | 126.7437 | 133.1691 | 1.0000 |  |
| 350.0-399.9 | 132.1401 | 127.9610 | 136.3192 | 1.0000 | |  | 350.0-399.9 | 127.4147 | 123.2774 | 131.5521 | 0.9972 |  |
| 400.0-449.9 | 133.1603 | 124.8203 | 141.5002 | 1.0000 | |  | 400.0-449.9 | 127.8259 | 118.8568 | 136.7949 | 1.0000 |  |
| 450.0-499.9 | 131.0027 | 127.4793 | 134.5261 | 0.9846 | |  | 450.0-499.9 | 128.7846 | 126.0739 | 131.4952 | 1.0000 |  |
| 500.0-549.9 | 136.3728 | 128.8928 | 143.8527 | 0.9991 | |  | 500.0-549.9 | 133.3995 | 124.4398 | 142.3592 | 1.0000 |  |
| 550.0-599.9 | 127.6929 | 115.7803 | 139.6055 | 0.9999 | |  | 550.0-599.9 | 128.7336 | 119.4089 | 138.0583 | 1.0000 |  |
| 600.0-649.9 | 133.1980 | 128.6144 | 137.7816 | 1.0000 | |  | 600.0-649.9 | 129.2377 | 125.9394 | 132.5359 | 1.0000 |  |
| 650.0-699.9 | 127.4809 | 114.6405 | 140.3213 | 1.0000 | |  | 650.0-699.9 | 141.7234 | 125.7531 | 157.6936 | 0.9437 |  |
| 700.0-749.9 | 133.0750 | 129.2199 | 136.9301 | 1.0000 | |  | 700.0-749.9 | 128.9790 | 126.1504 | 131.8076 | 1.0000 |  |
| 750.0-799.9 | 127.6747 | 114.8211 | 140.5284 | 1.0000 | |  | 750.0-799.9 | 149.8133 | 131.4319 | 168.1947 | 0.4594 |  |
| 800.0-849.9 | 142.6337 | 124.6135 | 160.6540 | 0.9978 | |  | 800.0-849.9 | 123.0489 | 111.6987 | 134.3991 | 0.9970 |  |
| 850.0-899.9 | NA | NA | NA | NA | |  | 850.0-899.9 | 125.2177 | 102.7548 | 147.6807 | 1.0000 |  |
| 900.0-949.9 | 129.3898 | 116.5351 | 142.2444 | 1.0000 | |  | 900.0-949.9 | 133.0074 | 122.2887 | 143.7262 | 1.0000 |  |
| 950.0-999.9 | 155.9363 | 134.0055 | 177.8671 | 0.5077 | |  | 950.0-999.9 | 133.7535 | 115.3725 | 152.1346 | 1.0000 |  |
| ≥1000.0 | 132.9630 | 124.0714 | 141.8546 | 1.0000 | |  | ≥1000.0 | 121.9095 | 111.2197 | 132.5993 | 0.9649 |  |
| urinary Na/K ratio |  |  |  |  | |  | urinary Na/K ratio |  |  |  |  |  |
| ＜3.0 | 129.2767 | 123.4827 | 135.0706 | Ref | | <0.001 | ＜3.0 | 125.1452 | 120.1032 | 130.1872 | Ref | <0.001 |
| 3.0-3.9 | 129.2777 | 126.0631 | 132.4924 | 1.0000 | |  | 3.0-3.9 | 126.6647 | 123.8618 | 129.4676 | 0.8817 |  |
| 4.0-4.9 | 131.2897 | 128.1846 | 134.3948 | 0.7910 | |  | 4.0-4.9 | 128.8873 | 126.1753 | 131.5993 | 0.2479 |  |
| 5.0-5.9 | 132.7820 | 129.6563 | 135.9077 | 0.3906 | |  | 5.0-5.9 | 130.5079 | 127.7947 | 133.2211 | 0.0496 |  |
| 6.0-6.9 | 134.1263 | 130.9299 | 137.3226 | 0.1552 | |  | 6.0-6.9 | 131.7169 | 128.9182 | 134.5157 | 0.0117 |  |
| 7.0-7.9 | 134.6127 | 131.0536 | 138.1718 | 0.1254 | |  | 7.0-7.9 | 131.5581 | 128.3560 | 134.7603 | 0.0232 |  |
| 8.0-8.9 | 134.4978 | 129.7051 | 139.2905 | 0.2464 | |  | 8.0-8.9 | 131.9264 | 127.2931 | 136.5598 | 0.0657 |  |
| ≥9.0 | 139.0629 | 129.5987 | 148.5270 | 0.1636 | |  | ≥9.0 | 129.5284 | 119.1394 | 139.9174 | 0.8217 |  |

BMI, body mass index; DBP, diastolic blood pressure; GEJE, Great East Japan Earthquake; LS means, least square means; METs, metabolic equivalents; SBP, systolic blood pressure.

Obesity was defined as BMI ≥ 25.0 kg/m^2^ according to world health organization criteria for Japanese individuals

The LS means of SBP was calculated by ANCOVA.

The multivariable-adjusted models included age (per 1-year increment), BMI (per 1-kg/m2 increment), drinking status (never-drinker, ex-drinker, and current drinker <23 g, 23.0–45.9 g, ≥46.0 g/day, and unknown), GGT (per 25.0-IU/L increment), estimated daily salt intake (per 1-g/day increment), estimated 24-h potassium excretion (per 5-mEq/day increment), smoking status (never-smoker, ex-smokers, current smoker [1-9, 10-19, ≥20 cigarettes a day and unknown), physical activity (per 50-METs-min/week increment), education status (below high school, vocational school or junior college or technical college, university or graduate school, and unknown), damage to the home during the GEJE (completely destroyed, large scale partial collapse, partially destroyed, no damage, do not live in the affected area, and unknown), and residential area (Miyagi and Iwate). To examine the association between SBP and urinary Na/K ratio, we analyzed the above multivariable adjusted model by replacing estimated salt intake and estimated 24-h potassium excretion with Na/K ratio.

For continuous variables, including age, BMI, estimated daily salt intake, potassium excretion, Na/K ratio, GGT, and leisure-time METs, we further calculated the p-values to analyze linear trends by scoring the categories and entering the number as a continuous term in the regression model.

The P for difference was calculated for each independent variables using Dunnett’s test with the reference categories being: age <31 years, BMI <19.0 kg/m2, never-drinker, GGT <25.0 IU/L, estimated daily salt intake <6.0 g/day, potassium excretion <20.0 mEq/day, Na/K ration < 3.0, never-smoker, physical activity <50 METs-min/week.
