# Supplementary figures and images for: Relationship between traditional risk factors for hypertension and systolic blood pressure in the Tohoku Medical Megabank Community-based Cohort Study
Source: Hypertens Res. 2024 Feb 29;47(6):1533–45. doi: 10.1038/s41440-024-01582-1 (PMC11150157; doi:10.1038/s41440-024-01582-1)

Supplemental Fig.1

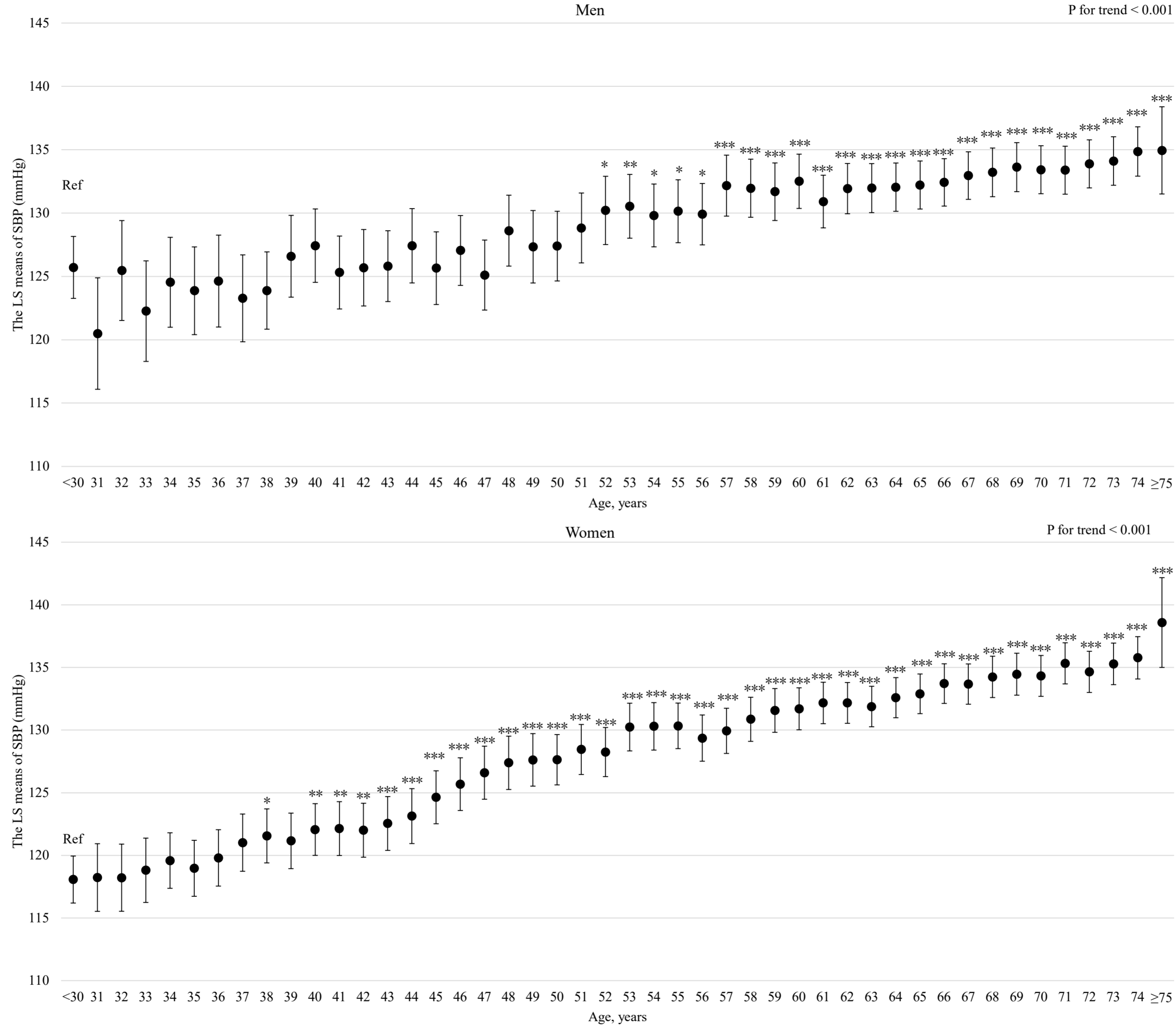

Supplement: Supplementary file 3 — Supplemental Figure 1 [file 41440_2024_1582_MOESM3_ESM.pdf]

Supplemental Fig.2

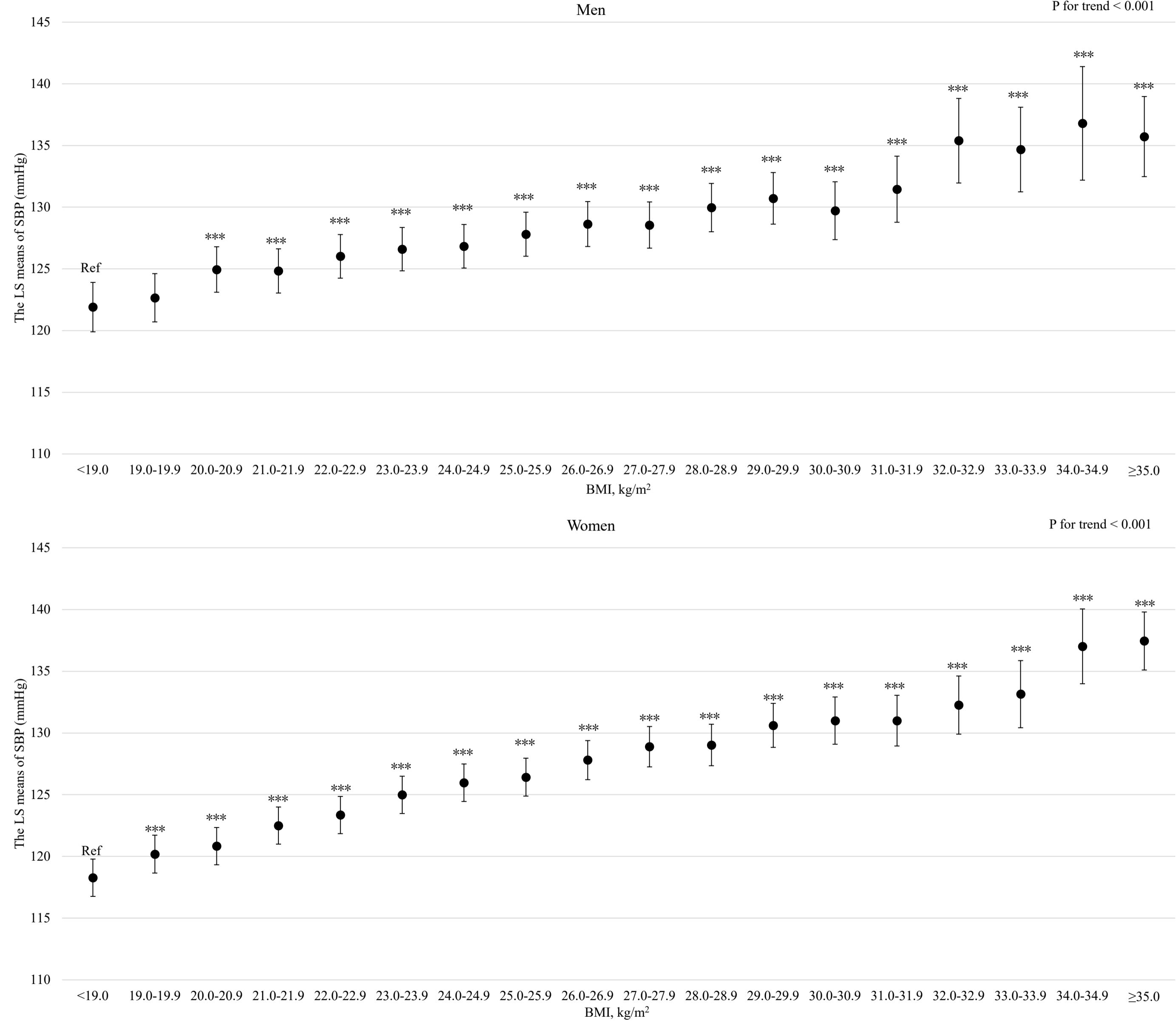

Supplement: Supplementary file 4 — Supplemental Figure 2 [file 41440_2024_1582_MOESM4_ESM.pdf]

Supplemental Fig.3

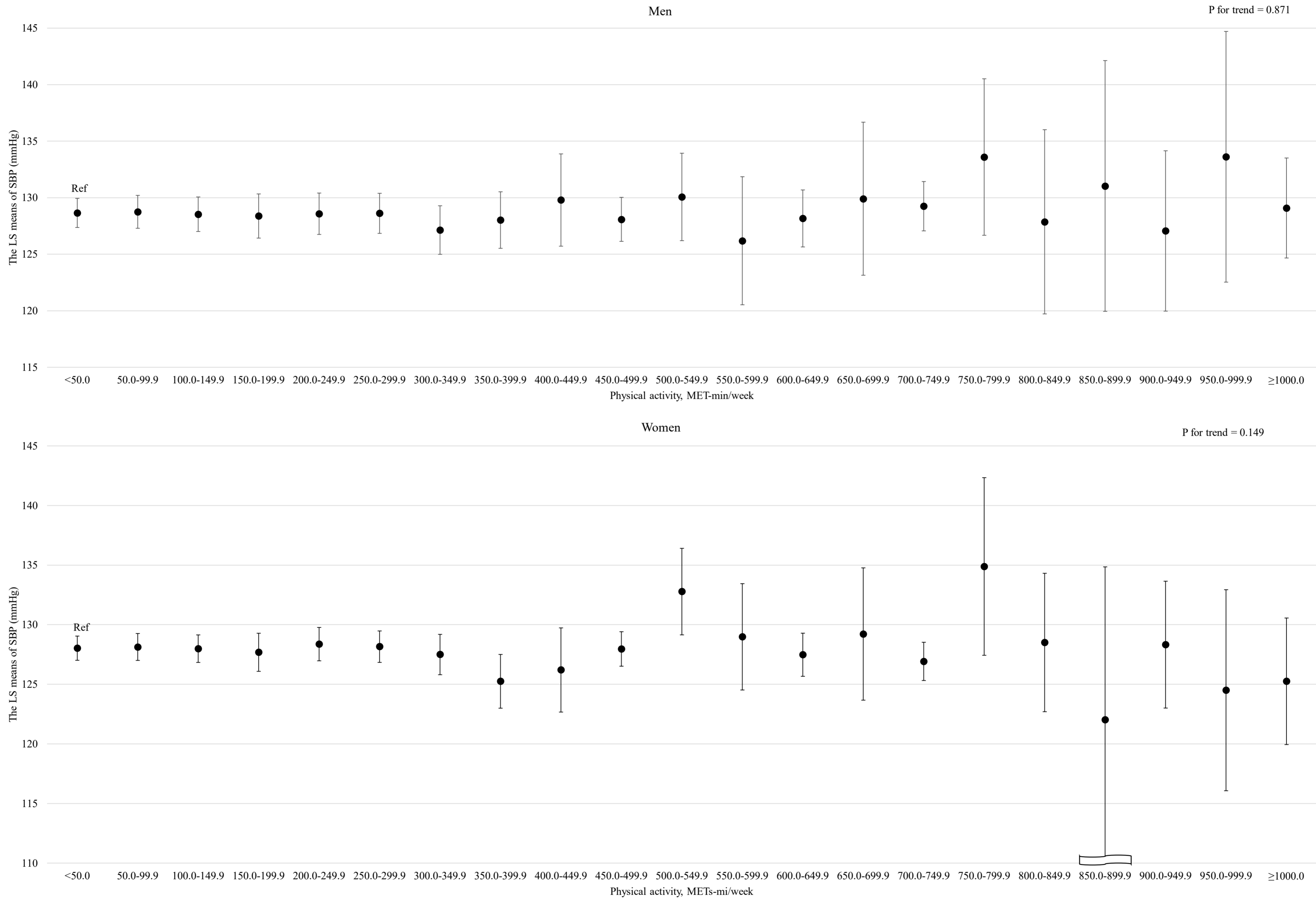

Supplement: Supplementary file 5 — Supplemental Figure 3 [file 41440_2024_1582_MOESM5_ESM.pdf]

Supplemental Fig.4

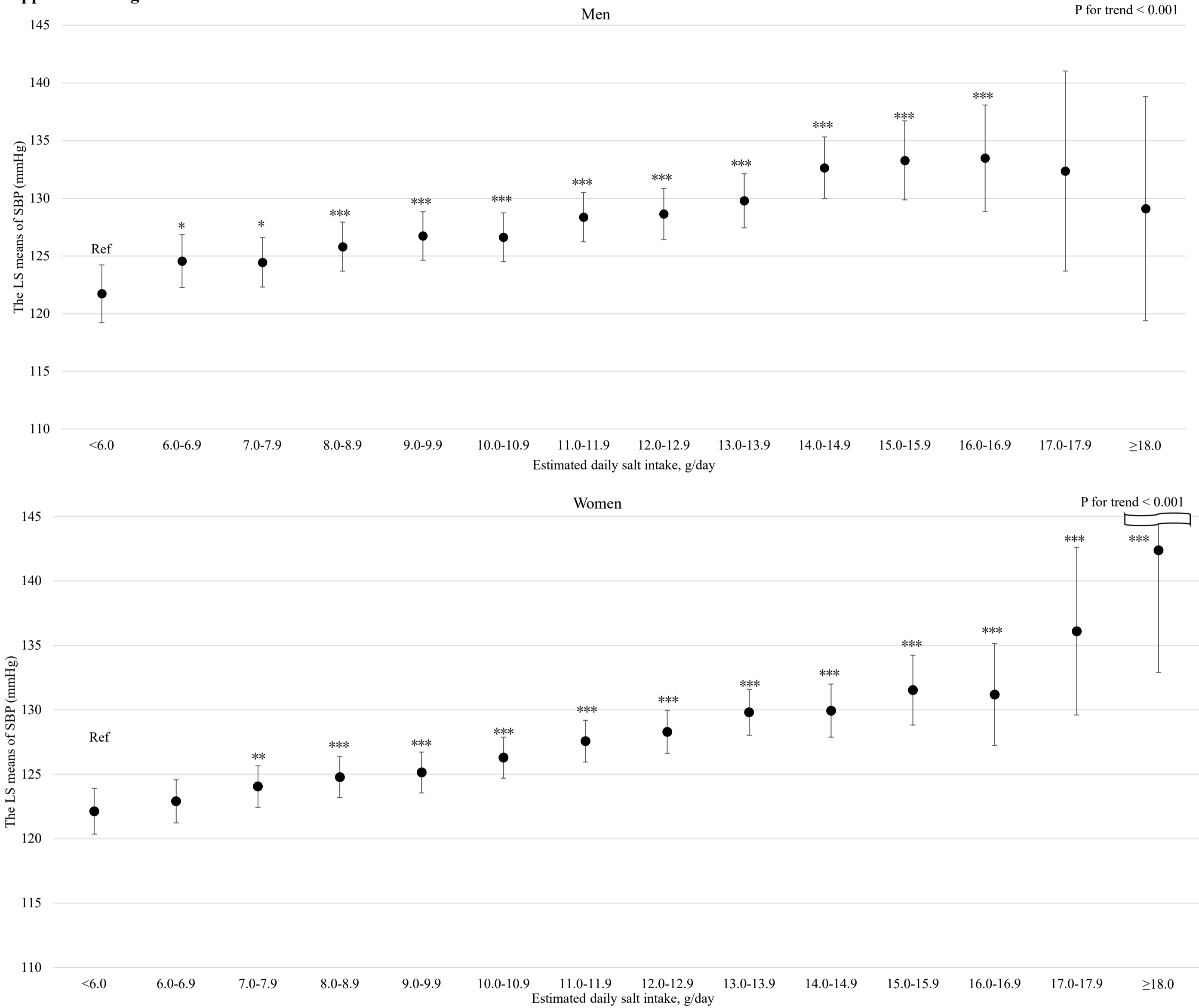

Supplement: Supplementary file 6 — Supplemental Figure 4 [file 41440_2024_1582_MOESM6_ESM.pdf]

Supplemental Fig.5

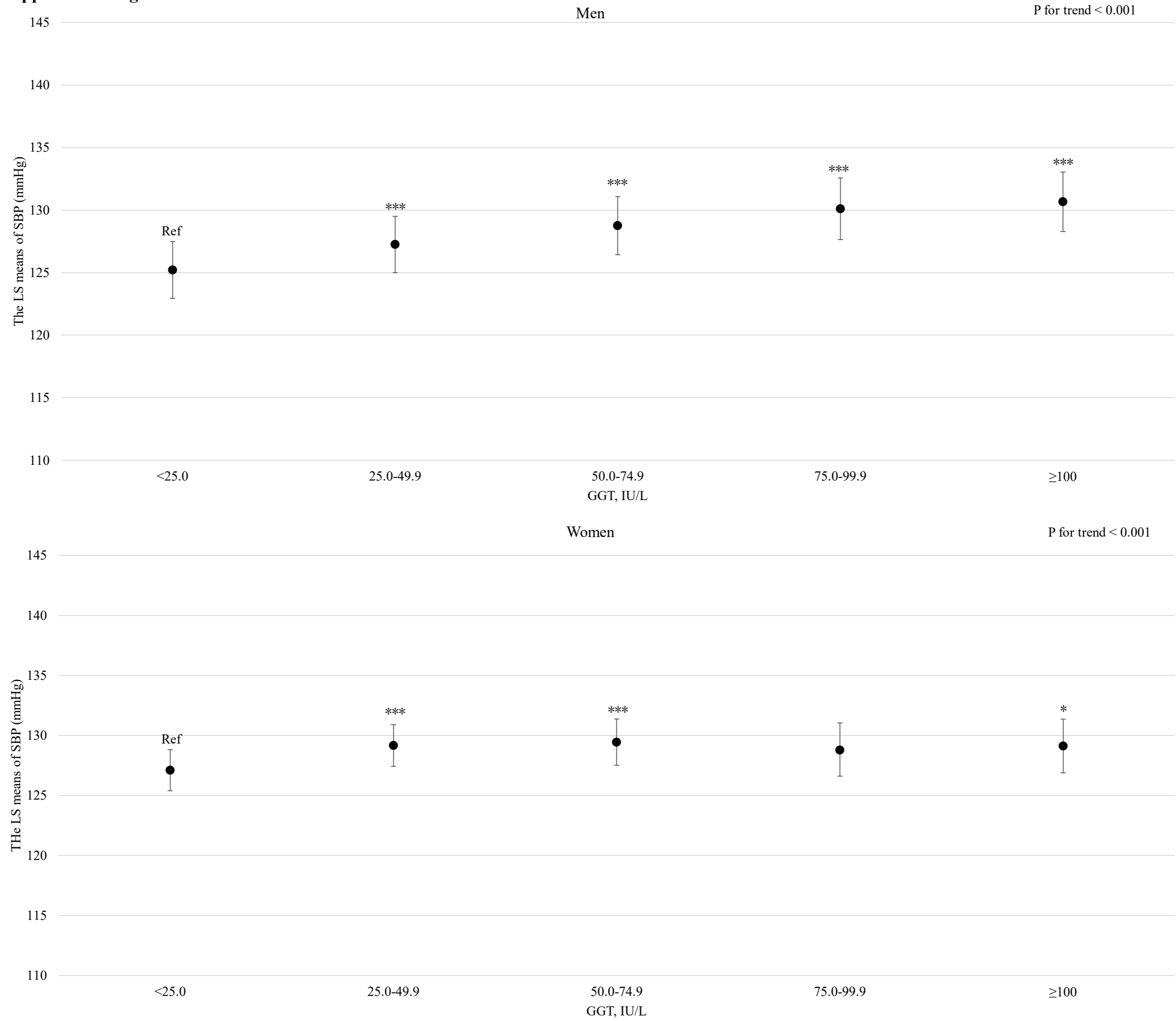

Supplement: Supplementary file 7 — Supplemental Figure 5 [file 41440_2024_1582_MOESM7_ESM.pdf]

Supplemental Fig.6

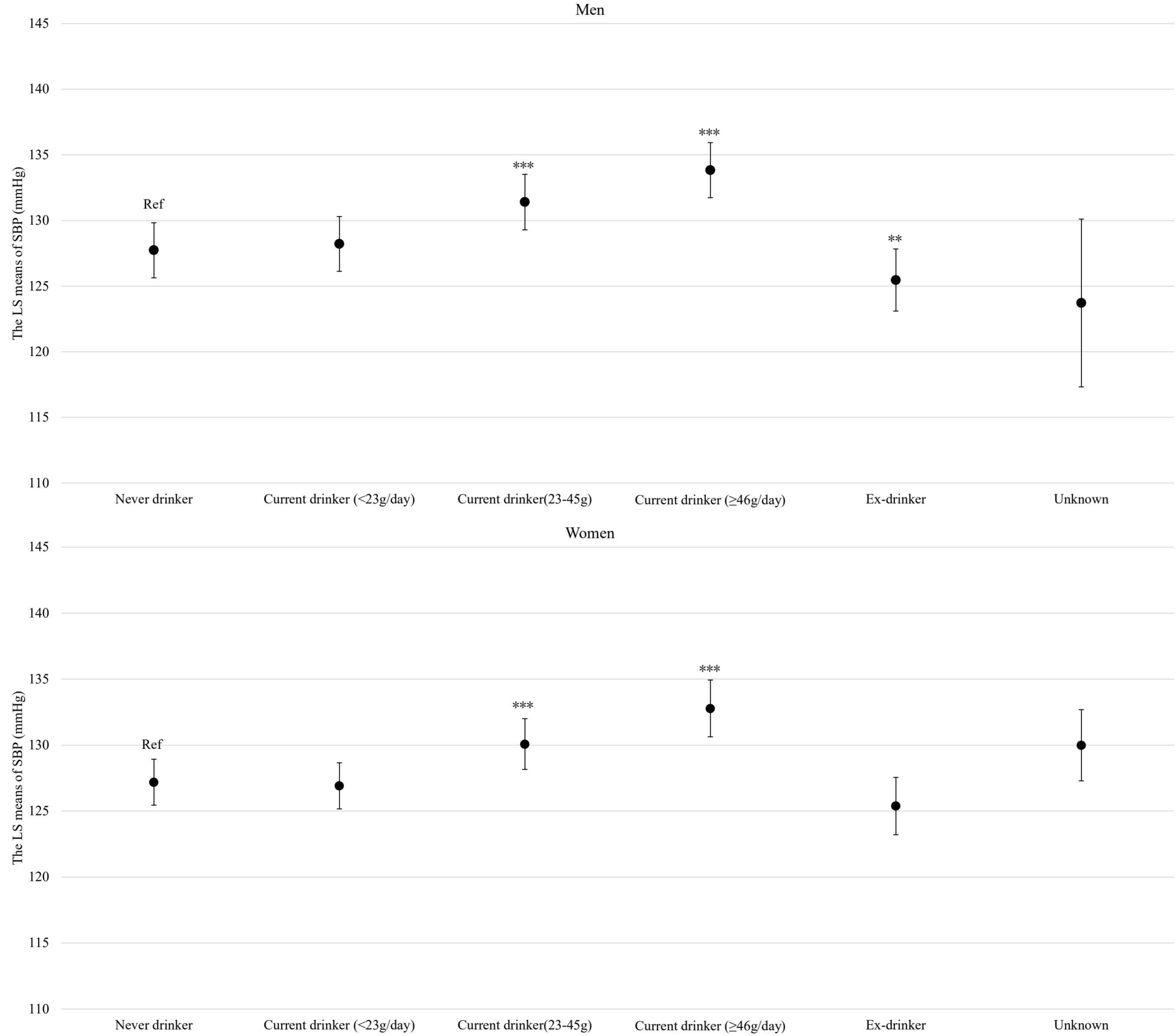

Supplement: Supplementary file 8 — Supplemental Figure 6 [file 41440_2024_1582_MOESM8_ESM.pdf]

Supplemental Fig.7

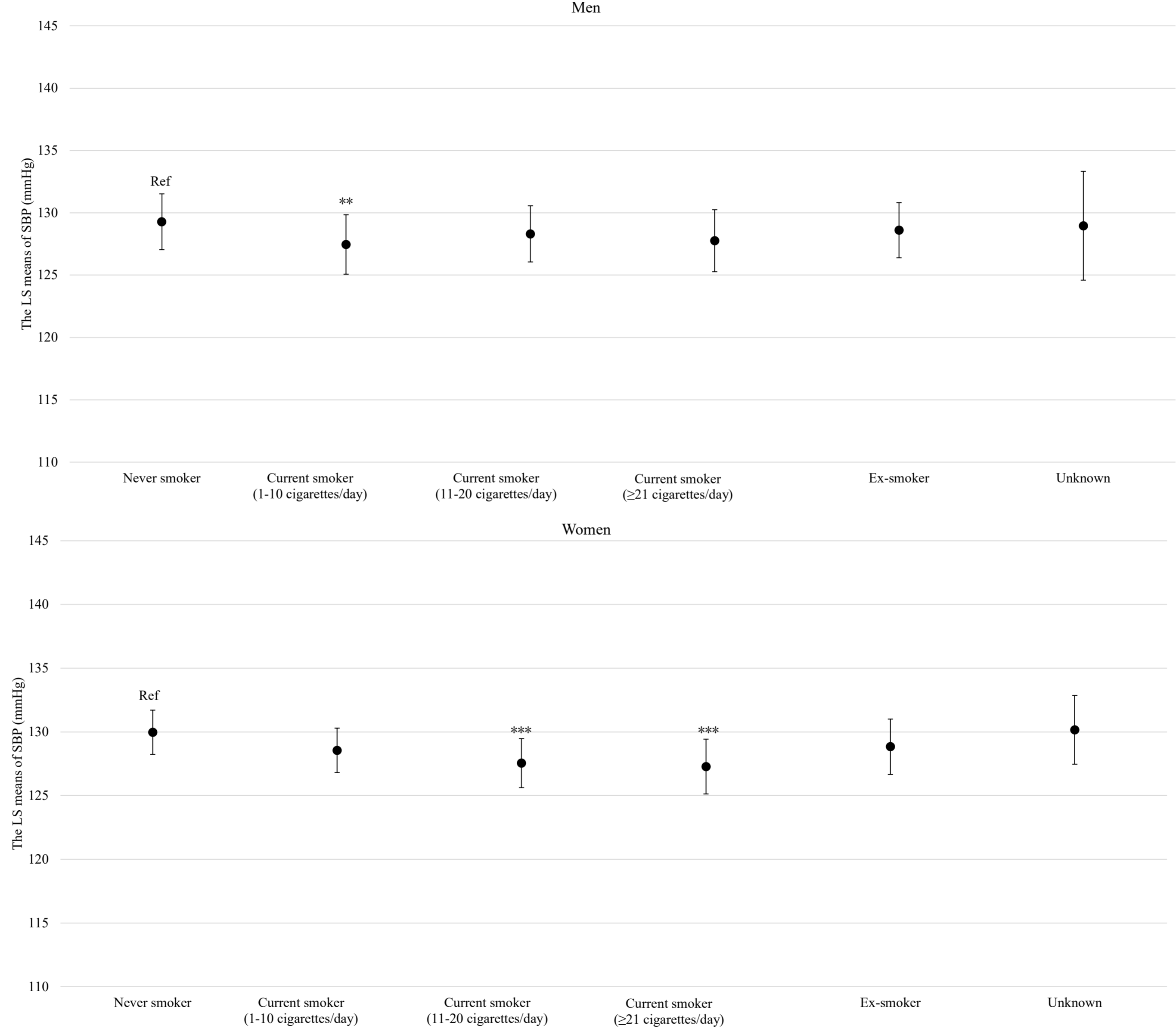

Supplement: Supplementary file 9 — Supplemental Figure 7 [file 41440_2024_1582_MOESM9_ESM.pdf]

Supplemental Fig.8

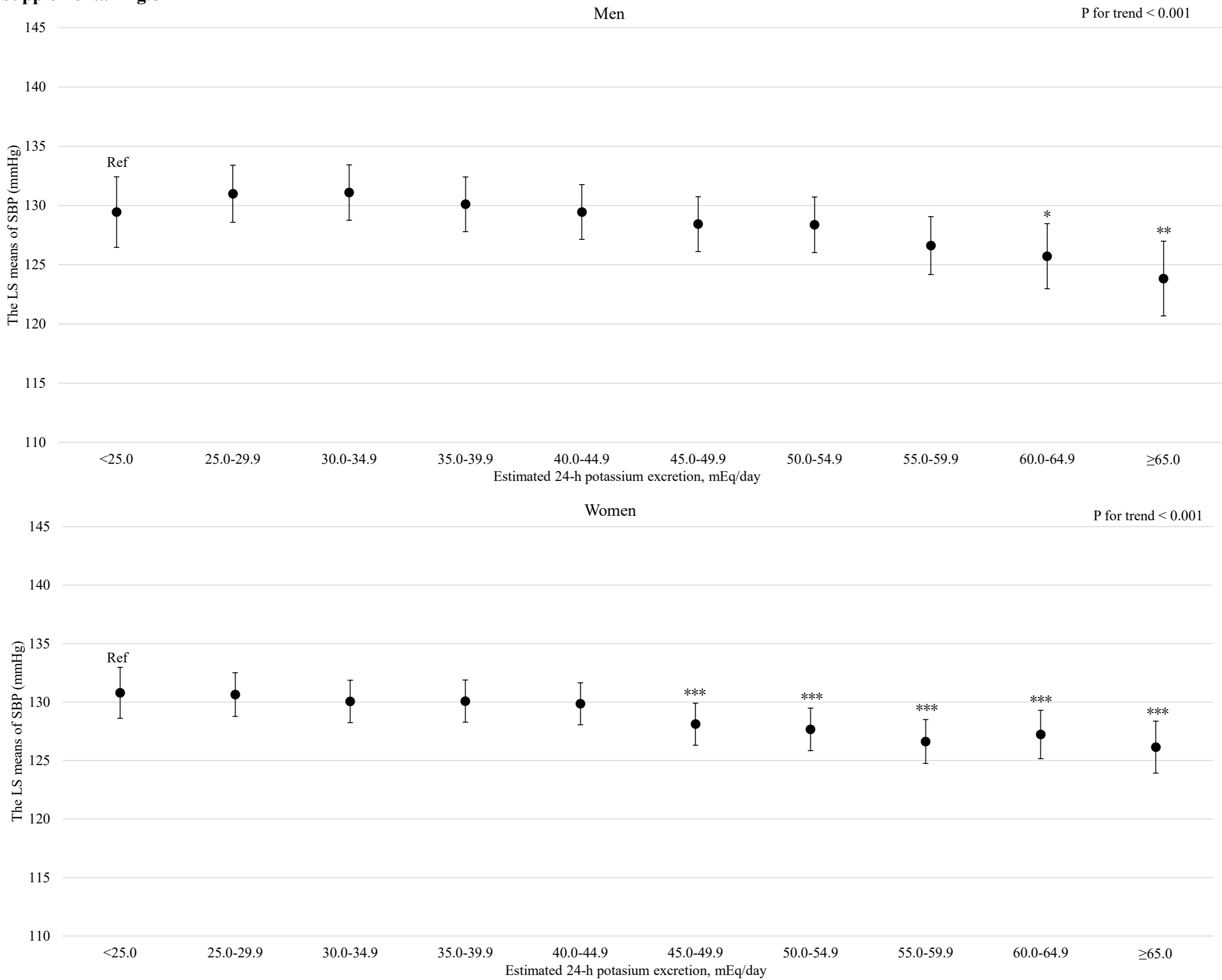

Supplement: Supplementary file 10 — Supplemental Figure 8 [file 41440_2024_1582_MOESM10_ESM.pdf]

Supplemental Fig.9

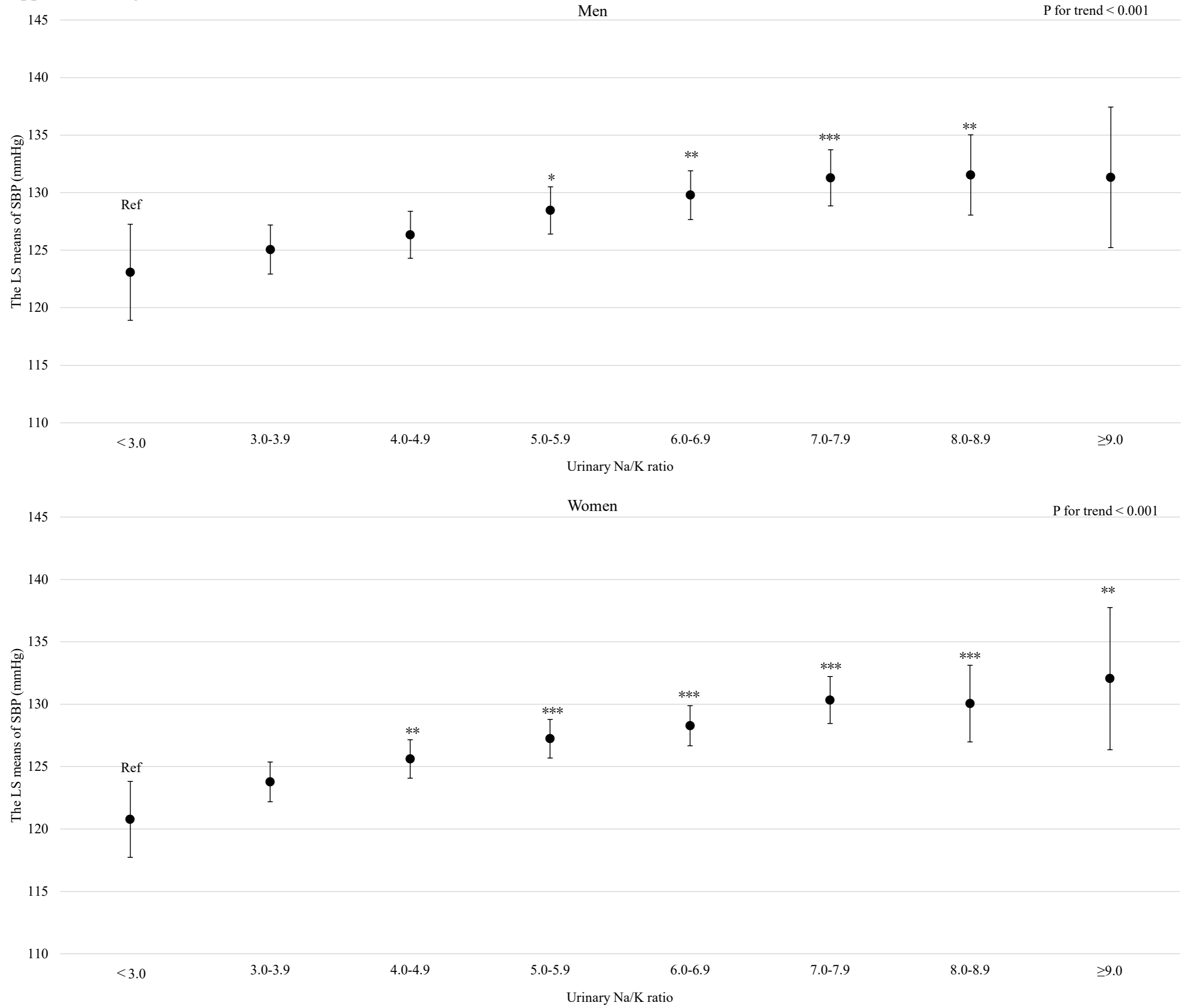

Supplement: Supplementary file 11 — Supplemental Figure 9 [file 41440_2024_1582_MOESM11_ESM.pdf]

Supplemental Fig.10

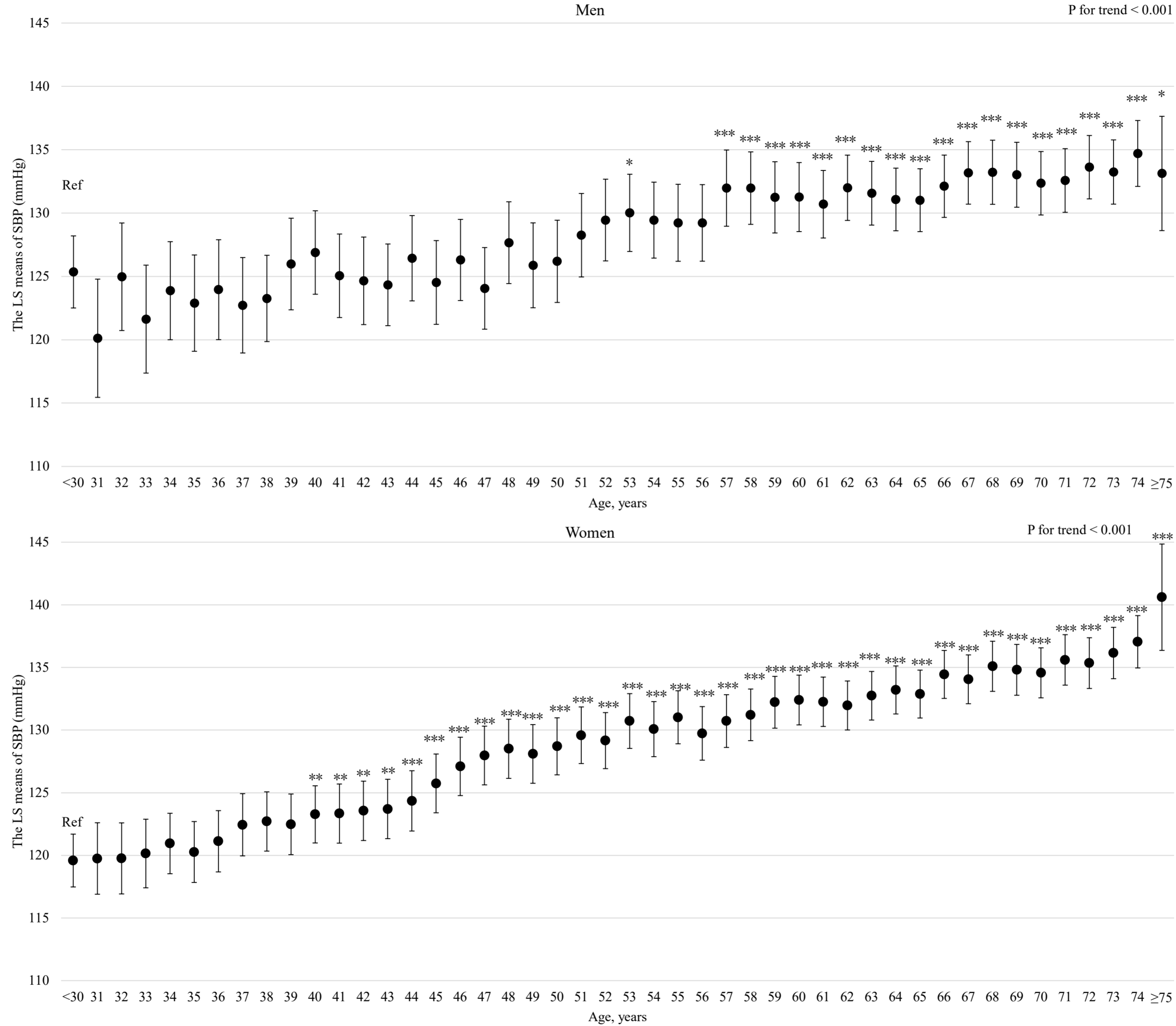

Supplement: Supplementary file 12 — Supplemental Figure 10 [file 41440_2024_1582_MOESM12_ESM.pdf]

Supplemental Fig.11

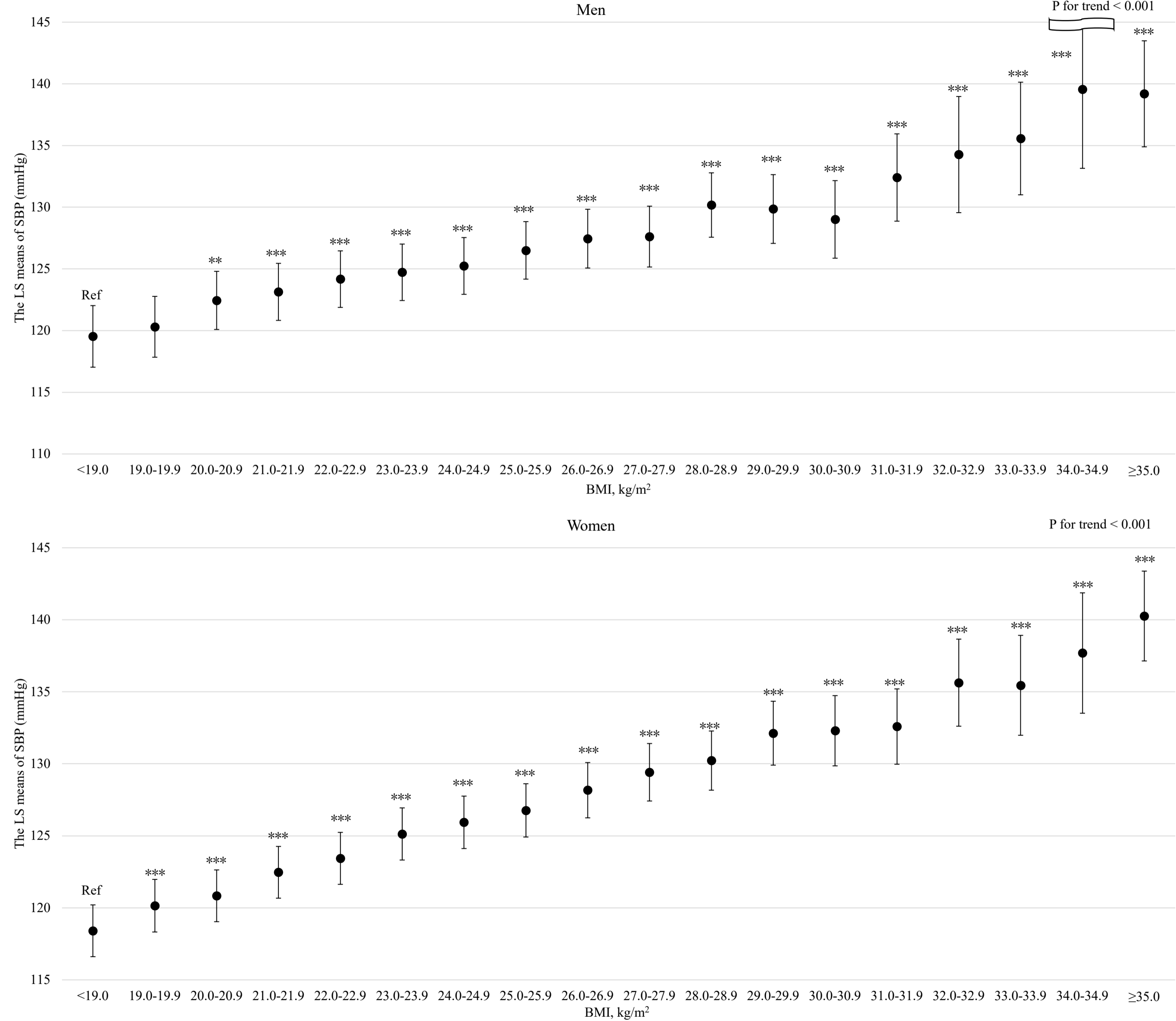

Supplement: Supplementary file 13 — Supplemental Figure 11 [file 41440_2024_1582_MOESM13_ESM.pdf]

Supplemental Fig.12

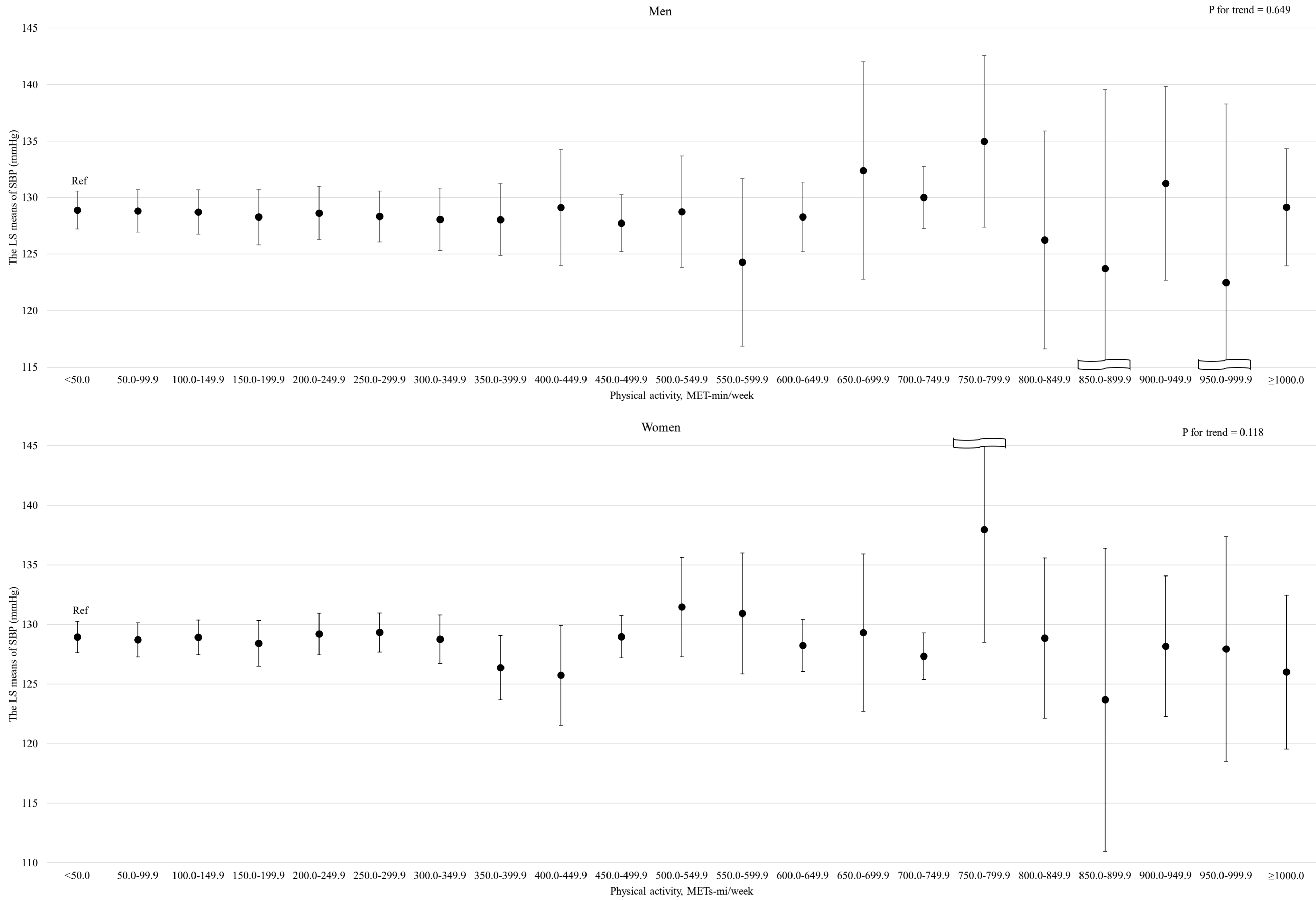

Supplement: Supplementary file 14 — Supplemental Figure 12 [file 41440_2024_1582_MOESM14_ESM.pdf]
