# Supplementary material for: Relationship between traditional risk factors for hypertension and systolic blood pressure in the Tohoku Medical Megabank Community-based Cohort Study
Source: Hypertens Res. 2024 Feb 29;47(6):1533–45. doi: 10.1038/s41440-024-01582-1 (PMC11150157; doi:10.1038/s41440-024-01582-1)
Supplement: Supplementary file 15 — Supplemental Figure Legend [file 41440_2024_1582_MOESM15_ESM.docx]

Supplemental Figure1. Association between age and SBP (in the model adjusted for BMI, estimated daily salt intake, GGT, drinking status, smoking status, estimated 24-h potassium excretion, physical activity, education status, damage to the home during the GEJE, and recruitment. p for difference was derived from Dunnett's test using age < 30 years as the reference. Bars represent 95% confidence intervals. p for difference showed as following: *p<0.05, **p<0.01, and ***p<0.001. p for trend was calculated by scoring the age categories and entering the number as a continuous term in the regression model. Abbreviations: BMI, body mass index; GGT, gamma-glutamyl transferase; GEJE, Great East Japan Earthquake; SBP, systolic blood pressure.

Supplemental Figure2. Association between BMI and SBP (in the model adjusted for age, estimated daily salt intake, GGT, drinking status, smoking status, estimated 24-h potassium excretion, physical activity, education status, damage to the home during the GEJE, and recruitment. p for difference was derived from Dunnett's test using BMI <19.0 kg/m^2^ as the reference. Bars represent 95% confidence intervals. p for difference showed as following: *p<0.05, **p<0.01, and ***p<0.001. p for trend was calculated by scoring the BMI categories and entering the number as a continuous term in the regression model. Abbreviations: BMI, body mass index; GGT, gamma-glutamyl transferase; GEJE, great east Japan earthquake; SBP, systolic blood pressure.

Supplemental Figure3. Association between physical activity and SBP (in the model adjusted for age, sex, BMI, estimated daily salt intake, GGT, drinking status, smoking status, estimated 24-h potassium excretion, education status, damage to the home during the GEJE, and recruitment). p for difference was derived from Dunnett's test using physical activity <50 MET-min/week as the reference. Bars represent 95% confidence intervals. p for difference showed as following: *p<0.05, **p<0.01, and ***p<0.001. p for trend was calculated by scoring the physical activity categories and entering the number as a continuous term in the regression model. Abbreviations: BMI, body mass index; GGT, gamma-glutamyl transferase; GEJE, great east Japan earthquake; METs, metabolic equivalents; SBP, systolic blood pressure.

Supplemental Figure 4. Association between estimated daily salt intake and SBP among participants without treatment for hypertension (in the model adjusted for age, BMI, GGT, drinking status, smoking status, estimated 24-h potassium excretion, physical activity, education status, damage to the home during the GEJE, and recruitment). p for difference was derived from Dunnett's test using estimated daily salt intake <6.0 g/day as the reference. Bars represent 95% confidence intervals. p for difference showed as following: *p<0.05, **p<0.01, and ***p<0.001. p for trend was calculated by scoring the estimated daily salt intake categories and entering the number as a continuous term in the regression model. Abbreviations: BMI, body mass index; GGT, gamma-glutamyl transferase; GEJE, Great East Japan Earthquake; SBP, systolic blood pressure.

Supplemental Figure 5. Association between GGT and SBP among participants without treatment for hypertension (in the model adjusted for age, BMI, estimated daily salt intake, drinking status, smoking status, estimated 24-h potassium excretion, physical activity, education status, damage to the home during the GEJE, and recruitment). p for difference was derived from Dunnett's test using GGT < 25.0 IU/L as the reference. Bars represent 95% confidence intervals. p for difference showed as following: *p<0.05, **p<0.01, and ***p<0.001. p for trend was calculated by scoring the GGT categories and entering the number as a continuous term in the regression model. Abbreviations: BMI, body mass index; GGT, gamma-glutamyl transferase; GEJE, Great East Japan Earthquake; SBP, systolic blood pressure.

Supplemental Figure 6. Association between drinking status and SBP among participants without treatment for hypertension (in the model adjusted for age, BMI, estimated daily salt intake, GGT, smoking status, estimated 24-h potassium excretion, physical activity, education status, damage to the home during the GEJE, and recruitment). p for difference was derived from Dunnett's test using never drinker as the reference. Bars represent 95% confidence intervals. p for difference showed as following: *p<0.05, **p<0.01, and ***p<0.001. Abbreviations: BMI, body mass index; GGT, gamma-glutamyl transferase; GEJE, Great East Japan Earthquake; SBP, systolic blood pressure.

Supplemental Figure 7. Association between smoking status and SBP among participants without treatment for hypertension (in the model adjusted for age, BMI, estimated daily salt intake, GGT, drinking status, estimated 24-h potassium excretion, physical activity, education status, damage to the home during the GEJE, and recruitment). p for difference was derived from Dunnett's test using never smoke as the reference. Bars represent 95% confidence intervals. p for difference showed as following: *p<0.05, **p<0.01, and ***p<0.001. Abbreviations: BMI, body mass index; GGT, gamma-glutamyl transferase; GEJE, Great East Japan Earthquake; SBP, systolic blood pressure.

Supplemental Figure 8. Association between estimated 24-h potassium excretion and SBP among participants without treatment for hypertension (in the model adjusted for age, BMI, estimated daily salt intake, GGT, drinking status, smoking status, physical activity, education status, damage to the home during the GEJE, and recruitment). p for difference was derived from Dunnett's test using estimated 24-h potassium excretion <25.0 mEq/day as the reference. Bars represent 95% confidence intervals. p for difference showed as following: *p<0.05, **p<0.01, and ***p<0.001. p for trend was calculated by scoring the estimated 24-h potassium excretion categories and entering the number as a continuous term in the regression model. Abbreviations: BMI, body mass index; GGT, gamma-glutamyl transferase; GEJE, Great East Japan Earthquake; SBP, systolic blood pressure.

Supplemental Figure 9. Association between urinary Na/K ratio and SBP among participants without treatment for hypertension (in the model adjusted for age, BMI, GGT, drinking status, smoking status, physical activity, education status, damage to the home during the GEJE, and recruitment). p for difference was derived from Dunnett's test using urinary Na/K ratio <3.0 as the reference. Bars represent 95% confidence intervals. p for difference showed as following: *p<0.05, **p<0.01, and ***p<0.001. p for trend was calculated by scoring the urinary Na/K ratio categories and entering the number as a continuous term in the regression model. Abbreviations: BMI, body mass index; GGT, gamma-glutamyl transferase; GEJE, Great East Japan Earthquake; SBP, systolic blood pressure.

Supplemental Figure 10. Association between age and SBP among participants without treatment for hypertension (in the model adjusted for BMI, estimated daily salt intake, GGT, drinking status, smoking status, estimated 24-h potassium excretion, physical activity, education status, damage to the home during the GEJE, and recruitment. p for difference was derived from Dunnett's test using age < 30 years as the reference. Bars represent 95% confidence intervals. p for difference showed as following: *p<0.05, **p<0.01, and ***p<0.001. p for trend was calculated by scoring the age categories and entering the number as a continuous term in the regression model. Abbreviations: BMI, body mass index; GGT, gamma-glutamyl transferase; GEJE, Great East Japan Earthquake; SBP, systolic blood pressure.

Supplemental Figure11. Association between BMI and SBP among participants without treatment for hypertension (in the model adjusted for age, estimated daily salt intake, GGT, drinking status, smoking status, estimated 24-h potassium excretion, education status, damage to the home during the GEJE, and recruitment. p for difference was derived from Dunnett's test using BMI <19.0 kg/m2 as the reference. Bars represent 95% confidence intervals. p for difference showed as following: *p<0.05, **p<0.01, and ***p<0.001. p for trend was calculated by scoring the BMI categories and entering the number as a continuous term in the regression model. Abbreviations: BMI, body mass index; GGT, gamma-glutamyl transferase; GEJE, great east Japan earthquake; SBP, systolic blood pressure.

Supplemental Figure 12. Association between physical activity and SBP among participants without treatment for hypertension (in the model adjusted for age, sex, BMI, estimated daily salt intake, GGT, drinking status, smoking status, estimated 24-h potassium excretion, education status, damage to the home during the GEJE, and recruitment). p for difference was derived from Dunnett's test using physical activity <50 MET-min/week as the reference. Bars represent 95% confidence intervals. p for difference showed as following: *p<0.05, **p<0.01, and ***p<0.001. p for trend was calculated by scoring the physical activity categories and entering the number as a continuous term in the regression model. Abbreviations: BMI, body mass index; GGT, gamma-glutamyl transferase; GEJE, great east Japan earthquake; METs, metabolic equivalents; SBP, systolic blood pressure.
